# PTPN2 targets TAK1 for dephosphorylation to improve cellular senescence and promote adipose tissue browning in T2DM

**DOI:** 10.3389/fphar.2023.1124633

**Published:** 2023-05-12

**Authors:** Yapeng Liu, Lu Han, Ping Zhu, Ming Song, Yaoyuan Zhang, Linlin Meng, Wei Zhang, Cheng Zhang, Ming Zhong

**Affiliations:** ^1^ The Key Laboratory of Cardiovascular Remodeling and Function Research, Chinese Ministry of Education, Chinese National Health Commission and Chinese Academy of Medical Sciences, The State and Shandong Province Joint Key Laboratory of Translational Cardiovascular Medicine, Department of Cardiology, Qilu Hospital, Cheeloo College of Medicine, Shandong University, Jinan, Shandong, China; ^2^ Department of General Practice, Qilu Hospital, Cheeloo College of Medicine, Shandong University, Jinan, Shandong, China

**Keywords:** PTPN2, TAK1, adipocytes, senescence, adipose tissue browning

## Abstract

**Introduction:** The energy imbalance when energy intake exceeds expenditure acts as an essential factor in the development of insulin resistance (IR). The activity of brown adipose tissue, which is involved in the dissipation of energy via heat expenditure decreases under type 2 diabetic mellitus (T2DM) state when the number of pathological aging adipocytes increases. Protein tyrosine phosphatase non-receptor type 2 (PTPN2) regulates several biological processes by dephosphorylating several cellular substrates; however, whether PTPN2 regulates cellular senescence in adipocytes and the underlying mechanism has not been reported.

**Methods:** We constructed a model of type 2 diabetic mice with PTPN2 overexpression to explore the role of PTPN2 in T2DM.

**Results:** We revealed that PTPN2 facilitated adipose tissue browning by alleviating pathological senescence, thus improving glucose tolerance and IR in T2DM. Mechanistically, we are the first to report that PTPN2 could bind with transforming growth factor-activated kinase 1 (TAK1) directly for dephosphorylation to inhibit the downstream MAPK/NF-κB pathway in adipocytes and regulate cellular senescence and the browning process subsequently.

**Discussion:** Our study revealed a critical mechanism of adipocytes browning progression and provided a potential target for the treatment of related diseases.

## Graphical Abstract

**Figure:**
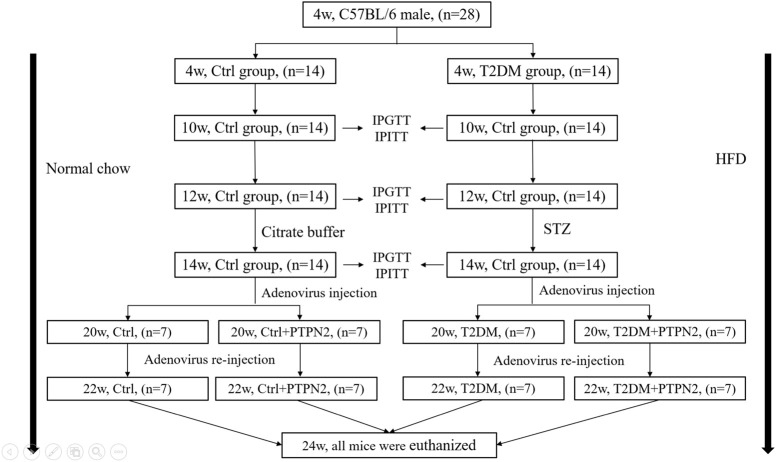
The timeline design of animal models.

## Introduction

Type 2 diabetes mellitus (T2DM) is rising to a global epidemic over the last few decades (Hildebrand et al., 2019). As a determining factor of T2DM, insulin resistance (IR) gains acceptance as the target of treatment (Sampath et al., 2019). Adipose tissue is a central regulator of systemic glucose homeostasis (Czech, 2020) and it can be subdivided into white adipose tissue (WAT), brown adipose tissue (BAT) and beige adipose tissue (Ying et al., 2017; Shimobayashi et al., 2018). WAT, including subcutaneous adipose tissue (SAT) and visceral adipose tissue (VAT), stores energy in the form of triglycerides while BAT drives thermogenesis by consuming fatty acids (Janssen et al., 2020; Krisko et al., 2020). Beige adipocytes are a kind of brown-like cells that have been identified in WAT. Both beige adipose tissue and BAT express high levels of uncoupled protein 1(UCP1) and are involved in the dissipation of energy via heat generation (Keipert et al., 2020; Xu et al., 2020). In T2DM, free fatty acids and pro-inflammatory mediators are highly released due to the imbalance between energy intake and output, eventually leading to the chronic low-grade inflammation and IR (Ljubkovic et al., 2019; Randeria et al., 2019). Thus, white adipose tissue browning ameliorates IR via the increase of energy output (Chen et al., 2021; Maliszewska and Kretowski, 2021). White adipose tissue browning is characterized by the gene expression of classical browning markers including UCP1, PRDM16, Dio2, PGC1α, and PPARα and when most of these markers are decreased, adipose tissue browning is inhibited (Miao et al., 2021). Adipose tissue browning can increase energy expenditure in humans and contribute to the control of adiposity in humans, as has been established in mice (Yoneshiro et al., 2011). Studies have shown that adipose tissue browning is inhibited in T2DM (Rogers et al., 2012; Moreno-Santos et al., 2016) and the corresponding mechanism needs to be further elucidated.

The role of senescence of adipose tissue in T2DM is well established (Börgeson et al., 2015; Gómez-Serrano et al., 2017). In tissues including BAT and WAT with senescence, free oxygen radical reactions are able to result in progressive accumulation of oxidative damage to DNA and lipids in addition to shortened telomeres, so may promote several pathological features (Jankovic et al., 2015; Wang and Hai, 2015). Mitochondrial dysfunction has been discussed in senescing brown adipocytes, where the release of reactive oxygen species (ROS) from dysfunctional mitochondria in senescing adipocytes results in lipid accumulation and chronic inflammation (Jankovic et al., 2015), and thus contributes the development of IR. Beige adipose tissue can reduce metabolic defects of WAT and is also influenced by aging (Xu et al., 2020; Cohen and Kajimura, 2021). Beige adipocytes are known to proliferate and differentiate from a sub-population of progenitors resident in white adipose tissue (Zoico et al., 2019). Besides, when metabolic changes are induced (e.g., nitrate-mediated activation), adipocyte browning can also occur in already differentiating WAT cells (Roberts, 2015). However, the defective ability of CD137/TMEM26 progenitor cells with strong differentiation potential has been proposed when trophic factors associated with regulating progenitor cell proliferation and differentiation in the adipose tissue microenvironment change during aging (Zoico et al., 2019), preventing the formation of beige adipose tissue. Adipose senescence in T2DM might be an important mechanism involved in the inhibition of adipose tissue browning.

The mitogen-activated protein kinase (MAPK) and nuclear factor-kappa B (NF-κB) pathway control cellular senescence traits by phosphorylating senescence-related proteins inorder to regulate their activity (Tian et al., 2019; Anerillas et al., 2020). In response to senescence stimulation, the MAPK/NF-κB pathway activates the P21/P53 axis, the P16 axis and the occurrence of senescence-associated secretory phenotype (SASP). The activated P38/MAPK-P16 pathway can induce senescence of progenitor cells and thereby inhibit adipose tissue browning (Berry et al., 2017). Transforming growth factor-activated kinase 1 (TAK1) is a critical upstream activator of the MAPK/NF-κB pathway (Dai et al., 2020; Zhao et al., 2021), which also triggers cellular senescence programs (Rajagopalan et al., 2014; Zhang et al., 2018a). However, whether TAK1 promotes cellular senescence and inhibits browning via the MAPK/NF-κB pathway in adipocytes remains unclear.

The protein tyrosine phosphatase non-receptor type 2 (PTPN2, gene ID: 19255) has extensive biological activity and acts as a crucial regulator of T2DM with its complications (Zhang et al., 2018b; Zheng et al., 2018). Our previous study has demonstrated that PTPN2 could improve diabetic nephropathy (Li et al., 2019). Nowadays, most studies about the effect of PTPN2 on adipose tissue are restricted to tissue level while the role of PTPN2 in regulating adipocytes browning directly needs to be further explained. PTPN2 has been demonstrated to be an inhibitor of P38/MAPK (Berry et al., 2017), however, whether PTPN2 improves adipocyte senescence by regulating TAK1/MAPK/NF-κB has not been reported.

In this study, we constructed a model of T2DM mice and demonstrated that PTPN2 could mediate adipose tissue browning by preventing adipose tissue from senescence, thereby improving IR. Mechanistically, we explored that PTPN2 targeted TAK1 directly for dephosphorylation to inhibit MAPK/NF-κB-mediated senescence for the first time.

## Materials and methods

### Animal models

We enrolled 28 C57BL/6 male mice aged 3 weeks weighting 14 (median)±2 g, purchased from experimental animal center of Shandong University, Ji’nan, China. All procedures were approved by Animal Care and Use Committee of Shandong University. Animals group-housed 3 per cage under standard laboratory conditions (25°C, 60%–70% relative humidity and 12 h of light and dark circulation) were tested for intraperitoneal glucose tolerance tests (IPGTT) after 1 week of adaptation.

After IPGTT, 28 mice were randomly assigned to the control group (Ctrl, *n* =14) and type 2 diabetes group (T2DM, *n* =14). The control group were fed a normal chow while the T2DM group received a high-fat diet (HFD, 34.5% fat, 17.5% protein, and 48% carbohydrate; Beijing HFK Bio-Technology, China). After 6 weeks, insulin tolerance was evaluated by intraperitoneal insulin tolerance tests (IPITT). After 8 weeks, IPITT and IPGTT were performed again to confirm the induction of IR. Mice exhibiting IR were given once low-dose of streptozotocin (STZ) injection intraperitoneally (Sigma, St. Louis, MO; 75–80 mg/kg i. p. in 0.1 mol/L citrate buffer, pH 4.5); the control group was administered with citrate buffer intraperitoneally only. IPGTT was measured again 2 weeks after injection of STZ. Most HFD/STZ-treated mice exhibited hyperglycemia (random blood glucose >11.1 mmol/L), IR and abnormal glucose tolerance.

At the age of 14 week, mice with similar degrees of hyperglycemia and body weight were randomly divided into the vehicle group (T2DM, *n* = 7) and PTPN2-overexpression group (T2DM + PTPN2, *n* = 7). Mice fed on a normal diet were classified as the non-diabetic control group, divided into the vehicle group (Ctrl, *n* = 7) and PTPN2-overexpression group (Ctrl + PTPN2, *n* = 7).

After food intervention, body weight was recorded every week and blood glucose level was examined every 2 weeks. The fasting glucose level was measured by OneTouch Glucometer (LifeScan, Milpitas, CA) following 6 h of fasting. IPGTT and IPITT were conducted according to the previously reported method (Li et al., 2019; Fukumura et al., 2021).

A recombinant pAdxsi adenovirus constitutively expressing PTPN2 systemically was constructed using the pAdxsi adenovirus system (SinoGenoMax, Beijing, China). Mouse-derived PTPN2 cDNAs were inserted into the pShuttle-CMV-EGFP vector and the pAdxsi vector adenovirus was used as a control vector. At 20 weeks mice were treated with 5 × 10^9^ plaque-forming units of virus by caudal intravenous injection. Adenovirus re-transferred at 22 weeks and the control group was injected with control virus (vehicle). All mice were euthanized at 24 weeks of age for further study as below.

### Histological and morphometric analysis of adipose tissue

Samples were taken from epididymal, subcutaneous and brown adipose tissue (SAT, VAT, and BAT) and each sample was cut into 2 parts on average, one of which was fixed with 4% paraformaldehyde, embedded with paraffin and sliced into 5um-thick sections. The morphology of adipocytes was analyzed from sections staining with hematoxylin and eosin (H&E). Adipocyte areas were assessed under X400 magnification within adipose tissue, and the number of adipocytes per area was obtained by quantitative morphometry with automated image analysis (Image-Pro Plus, Version 5.0; Media Cybernatics, Houston, TX) to assess the size of adipocytes.

### Immunohistochemical staining

Paraffin sections were treated with immunohistochemistry using microwave-based antigen retrieval method. These sections were incubated with primary rabbit polyclonal antibodies at 4°C overnight, and then with a matching biotinylated secondary antibody for 20 min at 37°C. The stained sections were developed with diaminobenzidin (DAB) and restained with hematoxylin. The staining results were observed under a confocal FV 1000 SPD laser scanning microscope (Olympus, Japan) and the integrated optical density values (IOD)/total IOD were evaluated by quantitative morphometry with automated image analysis (Image-Pro Plus, Version 5.0; Media Cybernatics, Houston, TX).

### Total protein extraction

Total protein of adipose tissue and cells was extracted using Cell Lysis (C2978, Sigma-Aldrich, USA) added with a protease inhibitor cocktail (1:100, CW2200S, CWBIO in Beijing, China). After centrifuging at 13,000×g, 4°C for 15 min, supernatant was collected and protein concentration was measured using BCA Protein Assay Kit (23227; ThermoFisher Scientific, United States). Then loading buffer was added, followed by incubating at 99°C for 5 min and subject to Western blotting analysis.

### Western blotting analysis

Proteins extracted from tissues or cells were separated by 10% as well as 12% SDS-PAGE and transferred on PVDF membrane. After incubation with 5% BSA (Albumin from bovine serum), transferred blots were incubated with primary antibodies (listed in Table 1) overnight at 4°C. On the next day, the PVDF membrane was added with anti-IgG horseradish peroxidase-conjugated secondary antibody at room temperature for 2 h. Blot images were detected by chemiluminescent reagent (WBKLS0500, Millipore, United States) via a luminescent image instrument (Amersham Imager 680, GE, United States). Total protein levels were normalized to β-actin levels.

**TABLE 1:** Antibodies and manufacturers.

Target antigen	Company	Catalog no.	Working concentration
PTPN2	Cell Signaling Technology	58935S	Western blotting: 1:1000
Normal rabbit IgG	Cell Signaling Technology	2729S	
β-actin	Abcam	ab8226	Western blotting: 1:1000
Myc	Cell Signaling Technology	2278S	Western blotting: 1:1000
			IF: 1:50
Flag	Sigma-Aldrich	F1804	Western blotting: 1:1000
			IF: 1:50
HA	ORIGENE	TA180128	Western blotting: 1:1000
Erk	Cell Signaling Technology	4695S	Western blotting: 1:1000
p-Erk	Cell Signaling Technology	4370S	Western blotting: 1:1000
JNK	Cell Signaling Technology	9252S	Western blotting: 1:1000
p-JNK	Cell Signaling Technology	4668S	Western blotting: 1:1000
P38	Cell Signaling Technology	9212S	Western blotting: 1:1000
p-P38	Cell Signaling Technology	4511S	Western blotting: 1:1000
P65	Cell Signaling Technology	8242S	Western blotting: 1:1000
p-P65	Cell Signaling Technology	3033S	Western blotting: 1:1000
IκB	Cell Signaling Technology	4812S	Western blotting: 1:1000
p- IκB	Cell Signaling Technology	2859S	Western blotting: 1:1000
P53	Cell Signaling Technology	2524S	Western blotting: 1:1000
			IHC:1:100
P16	Abcam	ab270058	Western blotting: 1:1000
			IHC:1:100
P21	Abcam	ab109199	Western blotting: 1:1000
P27	Abcam	ab32034	Western blotting: 1:1000
TAK1	Cell Signaling Technology	5206S	Western blotting: 1:1000
p-TAK1	Cell Signaling Technology	9339S	Western blotting: 1:1000
UCP1	Cell Signaling Technology	72298S	Western blotting: 1:1000
			IHC:1:100
PGC1α	Cell Signaling Technology	2178S	Western blotting: 1:1000
FABP4	Abcam	ab92501	Western blotting: 1:1000
INSR	Abcam	ab283689	Western blotting: 1:1000
Acox1	Abcam	ab2184032	Western blotting: 1:1000
PPARγ	Abcam	ab272718	Western blotting: 1:1000
SIRT3	Abcam	ab246522	Western blotting: 1:1000
SLC27A1	Abclonal	A12847	Western blotting: 1:1000
ATGL	Abcam	ab207799	Western blotting: 1:1000
			IHC:1:100
HSL	Abcam	ab45422	Western blotting: 1:1000
			IHC:1:100
LPL	Abcam	ab91606	Western blotting: 1:1000

### Total RNA extraction and quantitative RT-PCR assay

Total RNA was extracted from adipocytes using the Total RNA Extraction Kit (220011, Fastagen in Shanghai, China) based on protocols. The PrimeScript RT reagent kit was used for reversed-transcribtion including gDNA Eraser (RR047A, TaKaRa, Japan). The cDNA samples were subject to quantitative PCR process for indicators detection using the LightCycler^®^ 480 SYBR^®^ Green I Master (04887352001, Roche, Switzerland) by Roche LightCycler480 according to protocols. The level of β-actin was used for data normalization and the 2^−ΔΔCT^ method was used for calculation. Primer sequences are listed in Table 2.

**TABLE 2:** Primer sequences.

Gene name	Sequence 5′-3′
mouse *β-actin* forward	CCA​CAC​CCG​CCA​CCA​GTT​CG
mouse *β-actin* reverse	TAC​AGC​CCG​GGG​AGC​ATC​GT
mouse *Ptpn2* forward	AGC​AGT​GAG​AGC​ATT​CTA​CGG
mouse *Ptpn2 r*everse	GTG​CAG​AAA​GGT​GCT​GGG​TA
mouse *Ucp1 for*ward	TCA​CCA​CCC​TGG​CAA​AAA​CA
mouse *Ucp1 r*everse	GCA​GGT​GTT​TCT​CTC​CCT​GAA
mouse *Fabp4* forward	CGA​CAG​GAA​GGT​GAA​GAG​CAT​CAT​A
mouse *Fabp4* reverse	CAT​AAA​CTC​TTG​TGG​AAG​TCA​CGC​CT
mouse *Insr for*ward	GATCCGCGCCGCCTTTT
mouse *Insr r*everse	GGG​ACT​GTC​TCT​CGG​CTC​TC
mouse *Pgc1α for*ward	ACC​ATG​ACT​ACT​GTC​AGT​CAC​TC
mouse *Pgc1α r*everse	GTC​ACA​GGA​GGC​ATG​TTT​GAA​G
mouse *Atgl for*ward	TGA​CCA​TCT​GCC​TTC​CAG​A
mouse *Atgl r*everse	GAG​AGG​TTG​TTT​CGT​ACC​CA
mouse *Hsl for*ward	GCA​CTG​TGA​CCT​GCT​TGG​T
mouse *Hsl r*everse	CTG​GCA​CCC​TCA​CTC​CAT​A
mouse *Lpl for*ward	ACT​CTG​TGT​CTA​ACT​GCC​ACT​TCA​A
mouse *Lpl r*everse	ATA​CAT​TCC​CGT​TAC​CGT​CCA​T

### Elisa assay

The concentrations of IL6 (SM6000B, R&D) and IL1β (MHSLB00, R&D) of adipocyte supernatant was measured using Enzyme-linked immunosorbent assay (ELISA) kits according to instructions.

### Cell culture

Primary preadipocytes were isolated from SAT according to a standard protocol (Macotela et al., 2012). Then cells differentiated into mature adipocytes (hereafter primary adipocytes) exposed to serum-containing media supplemented with insulin, 1-methyl-3-isobutylxanthine (IBMX), indomethacin, dexamethasone, triiodothyronine (T3) and rosiglitazone and then switched to insulin, T3 and rosiglitazone for maintenance (Armani et al., 2014). Mouse 3T3-L1 cell line were purchased from the cellbank of Chinese Academy of Sciences. The mouse 3T3-L1 cell line is immortalised and does not undergo senescence as primary adipocytes. Then, cells were cultured and induced to differentiate (hereafter 3T3-L1-derived adipocytes) as previously reported (Molinari et al., 2021). After differentiation, Adipocytes were treated with tumor necrosis factor-alpha (TNFα) stimulation.

Human HEK293T cells and Hela cells were obtained from KeyGene BioTech (China) and cultured in complete Dulbecco’s Modified Eagle’s Medium (DMEM; C11995500BT, Gibco, United States) medium.

### Plasmids and siRNA transfection

Plasmids were transiently transfected into cells with the help of lipofectamine 3000 reagent (100022052, Invitrogen, USA) based on manufacturer’s protocols. The lipofectamine RNAiMAX reagent (56532, Invitrogen) was used for siRNA transfection. After plasmid or siRNA transfection for 24 h, cells could be treated for further processing, then lysed for protein collection. For adipocytes, siRNA and plasmids were transfected during initial plating and grown to confluence, and transfected once again after 48 h. After transfection, primary adipocytes and the 3T3-L1 cell line were differentiated into mature adipocytes. Plasmids are listed in Table 3.

**TABLE 3:** Plasmids and manufacturers.

Gene name	Tag	Vector name	Manufacturer
Vector only	C-Flag	pReceiver-M35	GeneCopoeia
Vector only	N-3xHA	pReceiver-M06	GeneCopoeia
Vector only	C-Myc	pReceiver-M09	GeneCopoeia
PTPN2	C-Flag	pReceiver-M35	GeneCopoeia
PTPN2	C-Myc	pReceiver-M09	GeneCopoeia
TRAF6	C-Flag	pReceiver-M35	GeneCopoeia
TAK1	C-Flag	pReceiver-M35	GeneCopoeia
TAK1	C-Myc	pReceiver-M09	GeneCopoeia
TAB1	N-3xHA	pReceiver-M06	GeneCopoeia
TAB2	C-Flag	pReceiver-M35	GeneCopoeia

### Co-immunoprecipitation (Co-IP) analysis

For exogenous Co-IP analysis, plasmids expressing PTPN2 and other indicators were transfected into HEK293T cells. After transfection with 24 h, cells were lysed with 1 mL Cell Lysis for protein extraction. After centrifugation for 15 min at 13,000g, 4°C, 100ul supernatant was removed as “Input” and the rest was incubated with agarose immunoprecipitation beads for tag combination with 4 °C incubation overnight on a rotary mixer. Beads were then washed for 5 times using Radio Immunoprecipitation Assay (RIPA) buffer (P0013c, Beyotime Biotechnology, China). Precipitated proteins were separated from beads after incubation at 99°C for 5min with 2x loading buffer. Before the incubation at 99°C, c-Myc peptide (M2435, sigma) or 3X-Flag peptide (F4799, sigma) was added to compete out the binding of c-Myc or 3X-Flag for necessary.

The protocol of endogenous Co-IP in 3T3-L1-derived adipocytes was similar to exogenous analysis described above. After supernatant preparation, corresponding antibodies were added for endogenous protein combination at 4°C for 1 h. Then protein A/G Plus Agarose (sc-2003, Santa Cruz Biotechnology, United States) was used for antibody-combination at 4°C overnight with gentle mixture. Notably, normal rabbit IgG (2729S, Cell Signaling Technology, United States) was used as a negative control (Ida et al., 2021).

### Laser confocal photography of cells

Hela cells were seeded on climbing slices for staining. Plasmids expressing PTPN2 and TAK1 were co-transfected into Hela cells for 24 h. After fixation, 0.1% Triton X-100 was used for cell membranes permeabilization for 5 min, and then 10% goat serum was used for blocking. Then cells were incubated using primary antibodies at 4°C overnight. On the next day, corresponding fluoresce secondary antibodies were used to multiple signaling cascades. DAPI was used for nuclei stain and mounting. Samples incubated with IgG and secondary antibody were used as negative control. Images were captured via laser confocal microscope or electric upright microscope.

### Senescence-associated β-galactosidase (SA-β-gal) staining

SA-β-gal staining was performed with Senescence β-Galactosidase Staining Kit (Beyotime, China) according to the manufacturer’s instruction. Primary adipocytes were fixed with β-Galactosidase fixing solution for 15 min after washed with PBS, and then maintained with the staining solution. The blue areas were recognized to be SA-β-gal-positive.

### Statistical analysis

All data were statistically analyzed using GraphPad Prism 8 (GraphPad, San Diego, CA) and expressed as mean ± standard deviation (SD). The number of replicates was indicated in figure legends (n-numbers) and we did independent biological repeats for each experiment. The Shapiro-wilk test was used to evaluate the normality assumption of the data distribution. For normally distribution, the statistical difference was determined by unpaired two-tailed Student’s t-tests, one-way ANOVA test or two-way ANOVA accordingly. If *p*-value was less than 0.05, the comparison was considered to be statistically significant. *P<0.05 , **P<0.01 , ***P<0.001 ; P#<0.05 , P##<0.01 , P###<0.001 .

## Results

### PTPN2 ameliorates cellular senescence in adipocytes

**FIGURE 1:**
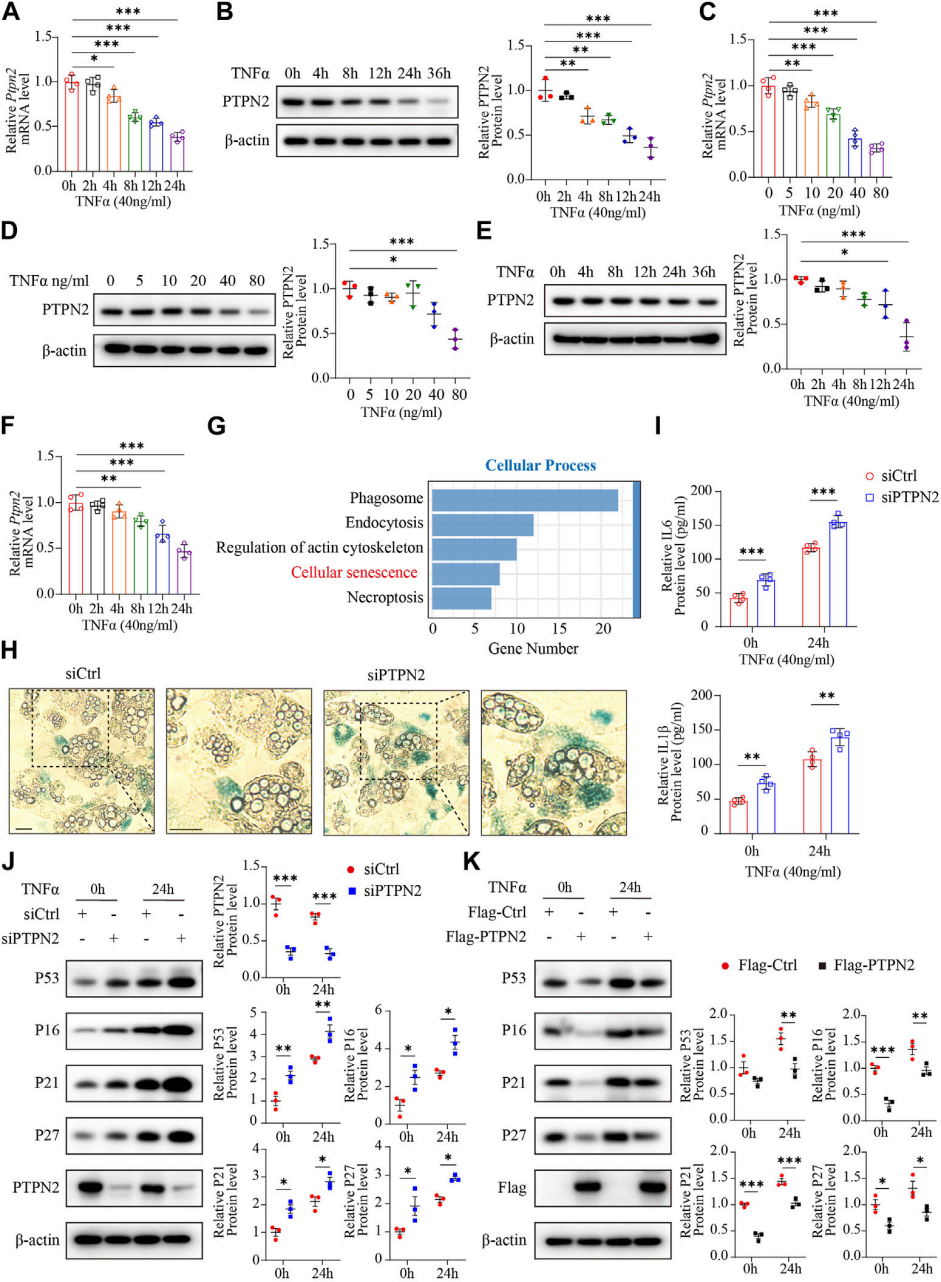
PTPN2 ameliorates cellular senescence in adipocytes. **(A)** Quantitative PCR (*n* = 4) of PTPN2 mRNA level in 3T3-L1-derived adipocytes treated with TNFα (40 ng/mL) for different time points. **(B)**, Western blotting of 3T3-L1-derived adipocytes treated with TNFα (40 ng/mL) for different time points (left) and data analysis (right, *n* = 3). β-actin was used for normalization. **(C),** Quantitative PCR (*n* = 4) of PTPN2 mRNA level in 3T3-L1-derived adipocytes treated with a concentration gradient of TNFα for 24 h. **(D)**, Western blotting of 3T3-L1-derived adipocytes treated with a concentration gradient of TNFα for 24 h (left) and data analysis (right, *n* = 3). **(E)**, Western blotting of primary preadipocyte-derived mature adipocytes treated with TNFα (40 ng/mL) for different time points (left) and data analysis (right, *n* = 3). **(F)**, Quantitative PCR (*n* = 4) of PTPN2 mRNA level in primary adipocytes treated with TNFα (40 ng/mL) for different time points. **(G)**, Primary preadipocytes were transfected with negative control siRNA (siCtrl) or PTPN2-siRNA (siPTPN2) and then induced to differentiate into mature primary adipocytes before TNFα stimulation for 24 h. The whole genome RNA-sequence analysis was performed and Kyoto Encyclopedia of Genes and Genomes (KEGG) enrichment analysis in cellular process between the two groups was shown (*n* = 3). **(H)**, The senescence‐associated β‐galactosidase (SA‐β‐gal) staining of primary adipocytes transfected with siCtrl or siPTPN2. Scale bar:50 μm. **(I)**, Elisa assay of interleukin-6 (IL6) and Interleukin-1β (IL1β) secreted from 3T3-L1-derived adipocytes with or without PTPN2 knockdown. Cells were pre-incubated with TNFα for 24 h before supernatant collection (*n* = 4). **(J)**, Western blotting images of indicated proteins in 3T3-L1-derived adipocytes transfected with negative control or PTPN2-siRNA in response to TNFα (40 ng/mL hereafter) and its quantitative analysis (*n* = 3). **(K)**, Western blotting images of indicated proteins in 3T3-L1-derived adipocytes transfected with or without Flag-tagged PTPN2 plasmid (Flag-PTPN2) overexpression in response to TNFα (*n* = 3). *P<0.05 , **P<0.01 , ***P<0.001 .

The expression level of PTPN2 in the serum is downregulated in T2DM patients (Zheng et al., 2018). As TNFα is a triggering factor in IR (Pedersen, 2017) and involved in several pathogenesis programs, we chose TNFα as the stimulation factor for adipocytes. 3T3-L1 cells were used to differentiate into mature adipocytes. Then, 3T3-L1-derived adipocytes were incubated with TNFα (40 ng/mL) for different time points (0, 4, 8, 12, 24, and 36 h) and we found that the mRNA and protein level of PTPN2 by TNFα stimulation was decreased in a time-dependent manner, especially significant after 24 h stimulation (Figures 1A, B). The mRNA and protein levels of PTPN2 also declined in a concentration-dependent manner in response to TNFα (Figures 1C, D) in 3T3-L1-derived adipocytes. Furthermore, the time-dependent downregulation was confirmed in mouse primary adipocytes (Figures 1E, F).

To further investigate the role of PTPN2 in adipocytes, a whole genome RNA-sequence analysis was performed using primary adipocytes with or without PTPN2 knockdown. Kyoto Encyclopedia of Genes and Genomes (KEGG) enrichment analysis in cellular processes revealed a strong association between PTPN2 and cellular senescence (Figure 1G). Therefore, we silenced the PTPN2 gene in primary adipocytes from mice using small interfering RNA (siRNA). PTPN2-deficient (siPTPN2) primary adipocytes exhibited an increase in senescence‐associated β‐galactosidase (SA‐β‐gal) activity compared with the control group (siCtrl) as demonstrated with SA‐β‐gal staining (Figure 1H). Interleukin-6 (IL6) and Interleukin-1β (IL1β) are two key components of SASP (Gorgoulis et al., 2019). Excessive IL6 and IL1β were secreted from 3T3-L1-derived adipocytes after TNFα stimulation and increased in siPTPN2 group (Figure 1I). Then, we tested the protein level of P53, P16, P27 and P21, which often gradually increase during cellular senescence. PTPN2 deficiency increased the level of senescence-related proteins (Figure 1J), and 3T3-L1-derived adipocytes with overexpressed PTPN2 confirmed these findings (Figure 1K). Thus, PTPN2 might alleviate senescent phenotype in adipocytes.

### PTPN2 inhibits phosphorylation of MAPK/NF-κB in adipocytes induced by TNFα

**FIGURE 2:**
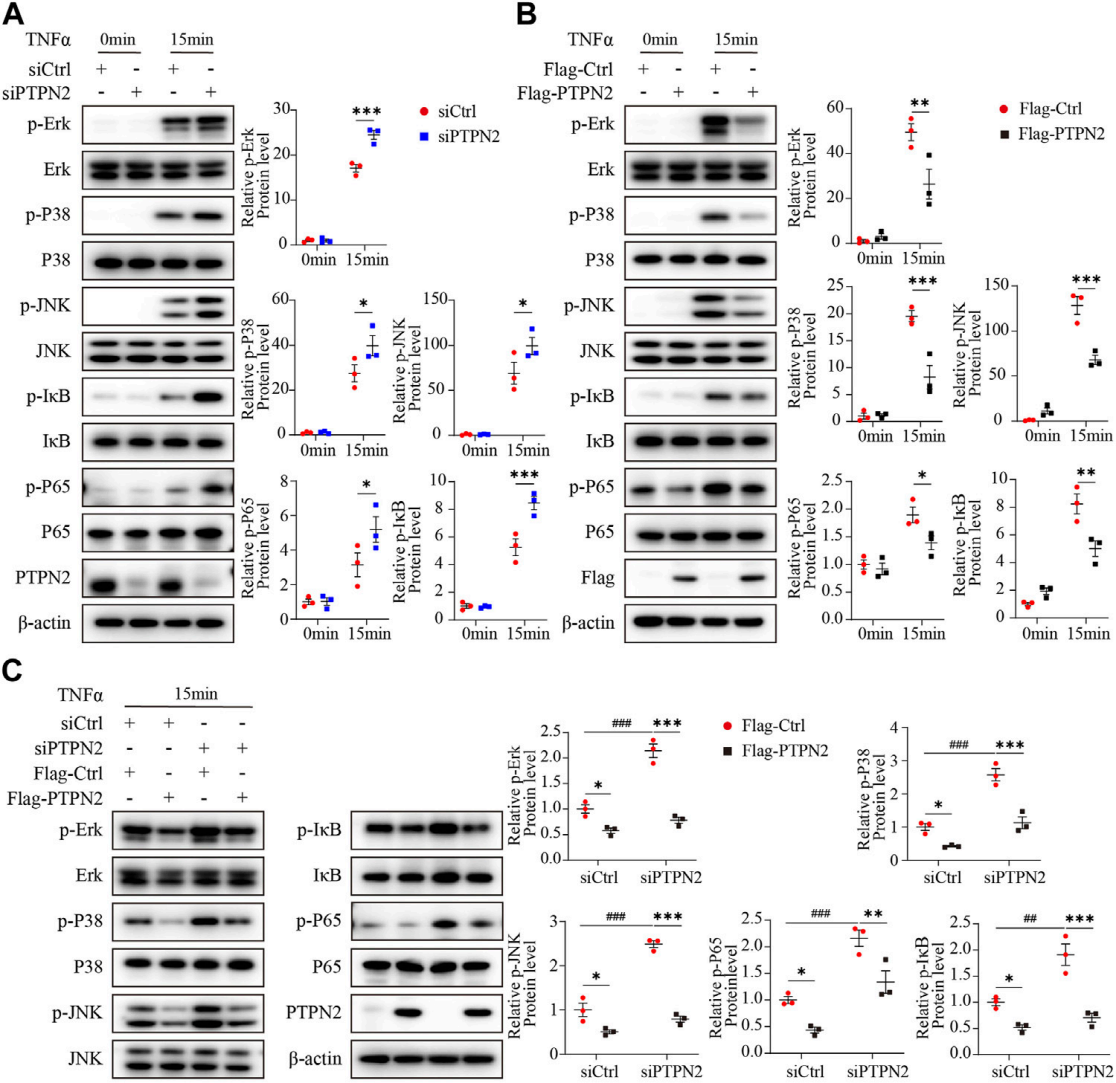
PTPN2 inhibits phosphorylation of MAPK/NF-κB in adipocytes induced by TNFα. **(A)**, Representative Western blotting images and data analysis of indicated proteins in 3T3-L1-derived adipocytes transfected with siCtrl or siPTPN2 with TNFα stimulation within 15 min (*n* = 3). **(B)**, Western blotting analysis of indicated proteins in 3T3-L1-derived adipocytes with or without Flag-PTPN2 overexpression in response to TNFα (*n* = 3). **(C)**, In 3T3-L1-derived adipocytes, PTPN2 protein level was silenced via siPTPN2 followed by overexpression with or without Flag-PTPN2 for rescue. Western blotting analysis of indicated proteins in response to TNFα was shown (*n* = 3). *P<0.05 , **P<0.01 , ***P<0.001 ; #P<0.05 , ##P<0.01 , ###P<0.001 .

As the MAPK/NF-κB pathway implements key traits of cellular senescence (Anerillas et al., 2020), we investigated the role of PTPN2 in the regulation of the MAPK/NF-κB pathway. We treated 3T3-L1-derived adipocytes with TNFα as an inducer and the phosphorylation level of Erk (p-Erk), P38 (p-P38), JNK (p-JNK), IκB (p- IκB) and P65 (p-P65) was upregulated in siRNA-mediated PTPN2-silent 3T3-L1-derived adipocytes after TNFα treatment (Figure 2A). Consistently, when 3T3-L1-derived adipocytes were transfected with Flag-tagged PTPN2 plasmid (Flag-PTPN2) for PTPN2 overexpression, the phosphorylation level of Erk, P38, JNK, IκB, and P65 was decreased significantly in response to TNFα (Figure 2B). To further confirm the effect of PTPN2 on MAPK/NF-κB pathway, we first silenced PTPN2 expression via siRNA, where the phosphorylation level was upregulated, and then rescued PTPN2 protein level by overexpression so that the phosphorylation level was inhibited subsequently (Figure 2C).

### PTPN2 interacts with TAK1 directly

**FIGURE 3:**
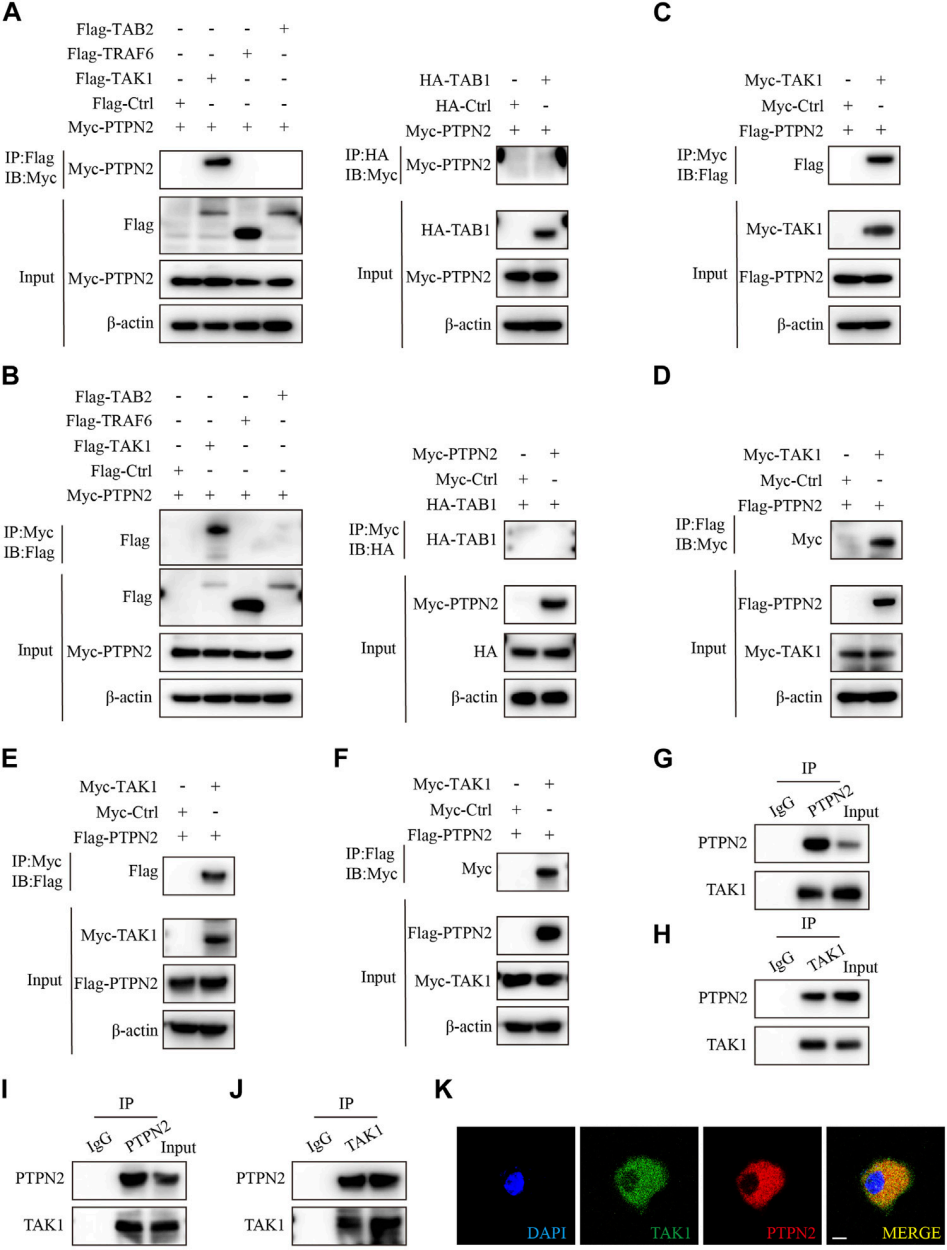
PTPN2 interacts with TAK1. **(A)**, Co-immunoprecipitation (Co-IP) analysis of HEK293T cells co-transfected with Myc-tagged PTPN2 and Flag-tagged TAK1, TRAF6, TAB2 to examine the interactors of PTPN2. Co-IP of whole cell lysates was immunoprecipitated using Flag beads (left). HEK293T cells were co-transfected with Myc-tagged PTPN2 and HA-tagged TAB1 and Co-IP of whole cell lysates was immunoprecipitated using HA beads (right). **(B)**, HEK293T cells were co-transfected with Myc-tagged PTPN2 and Flag-tagged TAK1, TRAF6, TAB2 and HA-tagged TAB1. Co-IP of whole cell lysates was immunoprecipitated using Myc beads. **(C, D)**, Co-IP assay of HEK293T cells co-transfected with Myc-TAK1 and Flag-PTPN2. Co-IP was performed using Myc beads **(C)** and Flag beads **(D)** respectively. **(E, F)**, Co-IP assay of Hela cells co-transfected with Myc-TAK1 and Flag-PTPN2. Co-IP was performed using Myc beads **(E)** and Flag beads **(F)** respectively. **(G, H)**, Western blotting images of indicated proteins of endogenous Co-IP in 3T3-L1-derived adipocytes immunoprecipitated with anti-PTPN2 **(G)**, anti-TAK1 **(H)** or with rabbit IgG control under TNFα stimulation. **(I, J)**, Western blotting images of indicated proteins of endogenous Co-IP in primary adipocytes immunoprecipitated with anti-PTPN2 **(I)**, anti-TAK1 **(J)** or with rabbit IgG control under TNFα stimulation. **(K)**, Representative confocal microscopic images of colocalization between PTPN2 and TAK1 in Hela cells co-transfected with Flag-tagged PTPN2 and Myc-tagged TAK1. Scale bar: 10 μm.

To identify exact molecules regulated by PTPN2, we co-transfected HEK293T cells with Myc-tagged PTPN2 plasmid and other plasmids encoding upstream regulators of MAPK/NF-κB pathway for the co-immunoprecipitation (Co-IP) experiment. PTPN2 interacted with TAK1 directly, but not with TRAF6, TAB2 and TAB1 (Figures 3A, B). Then, we confirmed the interaction between PTPN2 and TAK1 using tag-altered plasmids in HEK293T cells (Figures 3C, D) and Hela cells (Figures 3E, F), respectively. Endogenous Co-IP experiments in 3T3-L1-derived adipocytes (Figures 3G, H) and primary adipocytes (Figures 3I, J) confirmed the interaction between endogenous PTPN2 and TAK1. Moreover, we co-transfected PTPN2 and TAK1 plasmids into HeLa cells and confocal microscopy revealed that PTPN2 was colocalized with TAK1 (Figure 3K). Therefore, PTPN2 could bind to TAK1.

### PTPN2 inhibits the MAPK/NF-κB pathway via targeting TAK1 for dephosphorylation

**FIGURE 4:**
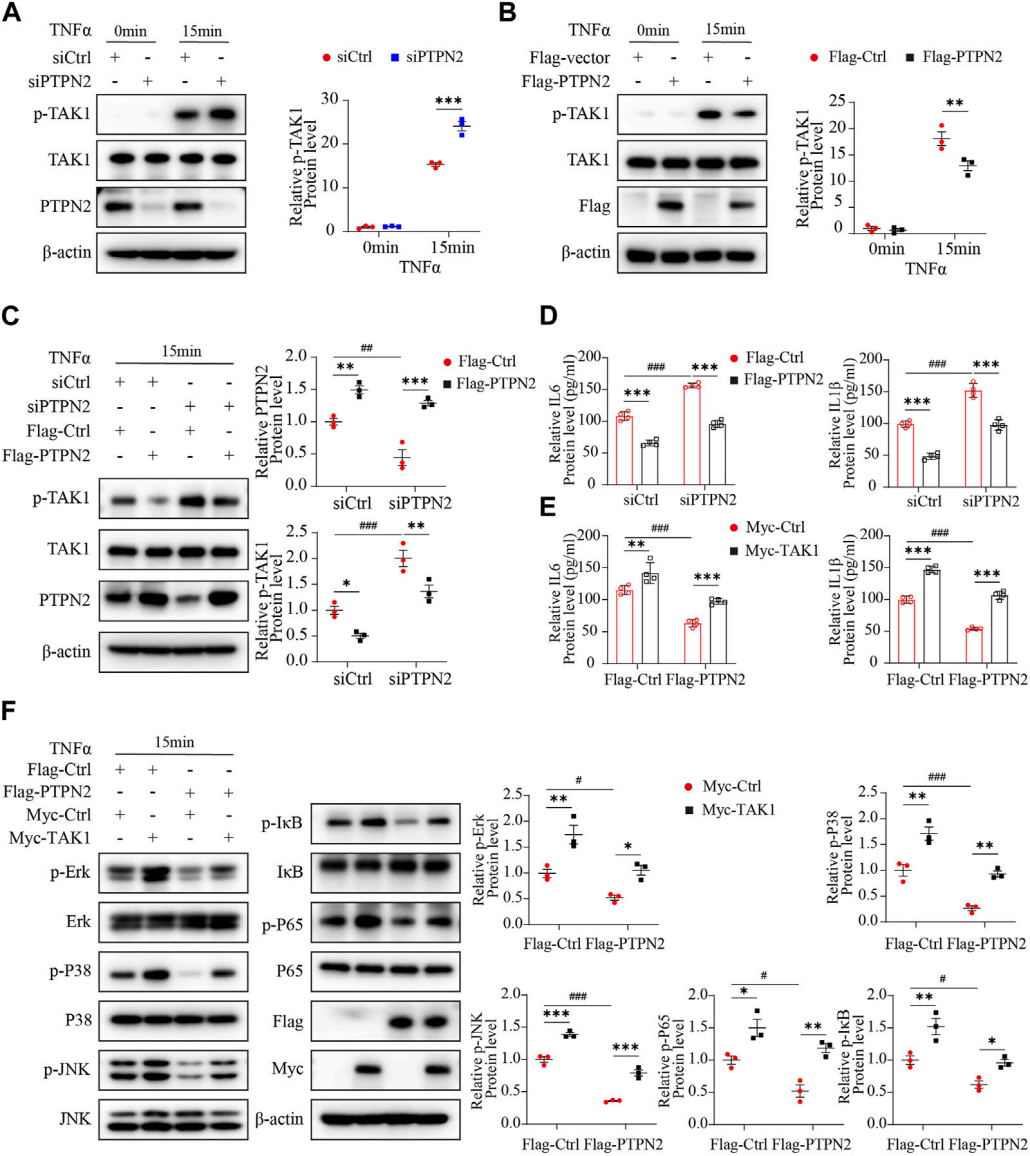
PTPN2 inhibits MAPK/NF-κB pathway via targeting TAK1 for dephosphorylation. **(A),** Western blotting images of indicated proteins in 3T3-L1-derived adipocytes transfected with siCtrl or siPTPN2 in response to TNFα (*n* = 3). **(B)**, Western blotting analysis of indicated proteins in 3T3-L1-derived adipocytes transfected with control vector or Flag-PTPN2 with TNFα stimulation (*n* = 3). **(C**, **D)**, In 3T3-L1-derived adipocytes, PTPN2 protein level was silenced via siPTPN2 firstly followed by overexpression with or without Flag-PTPN2 for rescue. Western blotting analysis of indicated proteins **(C),** (*n* = 3) and elisa assay of IL6 and IL1β secreted from 3T3-L1-derived adipocytes **(D),** (*n* = 4) in response to TNFα was shown. **(E, F)**, In 3T3-L1-derived adipocytes, PTPN2 was overexpressed via Flag-PTPN2 transfection followed by overexpression with or without Myc-TAK1. Elisa assay of IL6 and IL1β secreted from 3T3-L1-derived adipocytes **(E),** (*n* = 4) and Western blotting analysis of indicated proteins **(F)**, (*n* = 3) in response to TNFα was shown. *P<0.05 , **P<0.01 , ***P<0.001 ; P#<0.05 , P##<0.01 , P###<0.001 .

To explore the effect of PTPN2 on TAK1, we silenced PTPN2 in 3T3-L1-derived adipocytes using siRNA to test the phosphorylation level of TAK1. After the stimulation of TNFα, the level of phosphorylated TAK1 was upregulated in the PTPN2-deficient group compared with the control group (Figure 4A). Consistently, the phosphorylation level of TAK1 was significantly downregulated when PTPN2 was overexpressed in 3T3-L1-derived adipocytes (Figure 4B). Furthermore, we transfected Flag-tagged PTPN2 plasmid in 3T3-L1-derived adipocytes after PTPN2 silencing for rescue, and the result revealed that PTPN2-silence promoted the phosphorylation level of TAK1 while its phosphorylation was inhibited after PTPN2 overexpression (Figure 4C). A similar trend was also observed for the level of IL6 and IL1β secreted into the supernatant from cultured 3T3-L1-derived adipocytes (Figure 4D).

Finally, to investigate whether TAK1 has a role in the regulation of PTPN2 for the MAPK/NF-κB pathway, a recovery experiment was performed where TAK1 protein level was rescued in PTPN2-deficient 3T3-L1-derived adipocytes. Obviously, when stimulated with TNFα, the diminished SASP protein level of the PTPN2-overexpressed group was rescued by TAK1 overexpression (Figure 4E) and similar results were observed from the phosphorylation level detection of proteins of the MAPK/NF-κB pathway (Erk, P38, JNK, IκB and P65) (Figure 4F). Therefore, TAK1 acts as an essential regulator mediating the dephosphorylation effect of PTPN2 on the MAPK/NF-κB pathway.

### PTPN2 overexpression improves IR and lipid retention of adipose tissue in T2DM mice

The results above suggested that PTPN2 ameliorated adipocyte senescence and as adipocytes are the major cell type in adipose tissue, we proposed that PTPN2 could improve senescence in adipose tissue.

**FIGURE 5:**
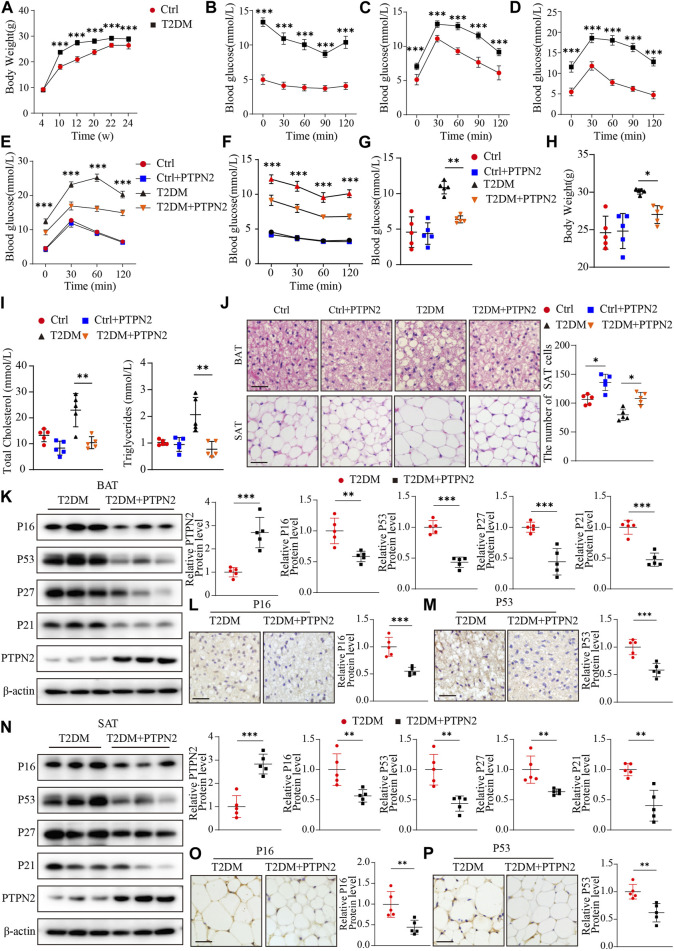
Overexpression of PTPN2 alleviates senescence in adipose tissue in T2DM mice. **(A),** Body weight of mice at the age of 4, 10, 12, 20, 22 and 24 weeks (*n* = 10). **(B),** Blood glucose level of IPITT at the age of 12 weeks (*n* = 10). **(C, D),** Blood glucose level of IPGTT at the age of 10 and 12 weeks (*n* = 10). **(E),** Blood glucose level of IPGTT in mice at the age of 24 weeks ( ***P<0.001 vs. T2DM + PTPN2, *n* = 5). **(F),** Blood glucose level of IPITT in mice at the age of 24 weeks ( ***P<0.001 vs. T2DM + PTPN2, *n* = 5). **(G)**, Fasting blood glucose level in mice at the age of 24 weeks (*n* = 5). **(H)**, Body weight of mice at the age of 24 weeks. **(I)**, Total Cholesterol level (left) and Triglycerides level (right) of mice at the age of 24 weeks. **(J),** BAT, SAT were stained with H&E (left) and the count of 3T3-L1-derived adipocytes in SAT within the field of the same size (right) was shown (*n* = 5, scale bar: 20 μm). **(K),** Representative Western blotting of indicated proteins in BAT of T2DM and T2DM + PTPN2 mice with data analysis (*n* = 5). **(L, M)**, Immunohistochemical staining of P16 (**L**) and P53 (**M**) protein in BAT, respectively (brown staining considered to be positive; scale bar: 20 μm). **(N),** Representative Western blotting of indicated proteins in SAT of T2DM and T2DM + PTPN2 mice with data analysis (*n* = 5). **(O, P)**, Immunohistochemical staining of P16 (**O**) and P53 (**P**) protein in SAT, respectively. scale bar: 20 μm. *P<0.05 , **P<0.01 , ***P<0.001 .

A non-genetic rodent model closely resembling human T2DM was generated by giving male C57BL/6 mice HFD as well as the STZ treatment (referred to as T2DM hereafter) while normal-diet fed mice served as controls (Ctrl hereafter). Multiple metabolic parameters of mice were examined to confirm the model generation. The body weight gain in the T2DM group was significantly increased compared with that in the control group after week-10 as expected (Figure 5A). IR in mice was verified by IPITT which showed that the blood glucose levels of T2DM group were higher than that of the control group at week-12 (Figure 5B). IPGTT was also tested at the age of 10 and 12 weeks, showing that blood glucose levels in the T2DM group were significantly elevated compared to the control group at all of the 5 time points (Figures 5C, D). Therefore, the T2DM model induced by HFD and STZ showed typical features of IR, hyperglycaemia and obesity, resembling the state of human T2DM.

For the T2DM model, IPGTT and IPITT demonstrated that compared with the control group, blood glucose levels in T2DM group were higher at all of the points tested at the age of 24 weeks, while levels in the PTPN2-overexpression group (T2DM + PTPN2) were lower than those in T2DM mice, indicating an improvement role of PTPN2 in IR and hyperglycaemia (Figures 5E, F). The fasting blood glucose level also showed that PTPN2 overexpression improved hyperglycaemia in T2DM mice (Figure 5G). We also discovered that the body weight, total cholesterol level and triglycerides level were decreased in the PTPN2-overexpression group compared with that in the T2DM group (Figures 5H, I). As adipose tissue plays a pivotal role in the development of insulin resistance, we isolated BAT, SAT and VAT respectively for subsequent experiments. The H&E staining demonstrated that the number of adipocytes in SAT counted in the T2DM group was lower than that of the control group in a field of identical size, indicating the bigger size and more lipid content of adipocytes in T2DM group. However, after Ad-PTPN2 treatment, the adipocyte number of SAT in the T2DM + PTPN2 group was significantly higher than that in T2DM group and a similar trend was also observed in VAT (data not shown). For BAT, we found that there were unilocular adipocytes akin to white adipocytes in T2DM mice while after PTPN2 intervention, there was a switch from unilocular to multilocular morphology in the T2DM + PTPN2 group (Figure 5J).

### Overexpression of PTPN2 alleviates senescence in adipose tissue in T2DM mice

We have demonstrated that PTPN2 ameliorated cellular senescence in adipocytes *in vitro*, then we assessed the effects of PTPN2 on adipose tissue *in vivo* and proteins related to senescence were analyzed in T2DM mice. Western blotting showed that the protein levels of P16, P53, P27 and P21 was downregulated in BAT of the PTPN2-overexpressing mice compared with the T2DM group without PTPN2-overexpression (Figure 5K). Furthermore, two main indicators, P16 and P53, were characterized by immunohistochemical staining in adipose tissue which showed that the protein level of p16 and P53 in the T2DM + PTPN2 group after PTPN2 intervention was significantly decreased compared with the T2DM group (Figures 5L, M). Similar results were also obtained from SAT of T2DM mice, indicating the role of PTPN2 in improving senescence in adipose tissue (Figures 5N–P).

### PTPN2 promotes the browning process of adipocytes in adipose tissue

**FIGURE 6:**
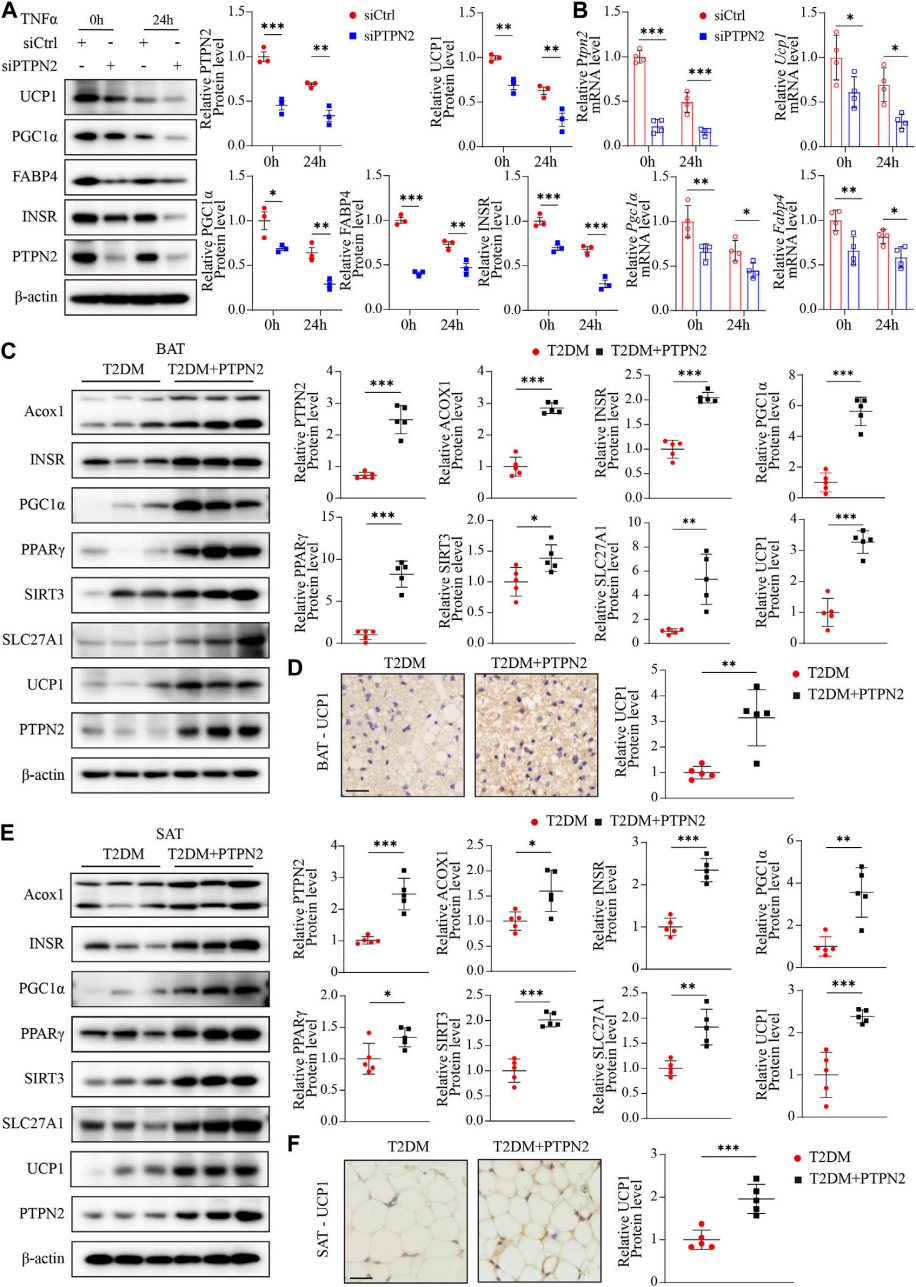
PTPN2 promotes the browning process of adipocytes in adipose tissue. **(A),** Western blotting analysis of indicated proteins in 3T3-L1-derived adipocytes transfected with siCtrl or siPTPN2 in response to TNFα (*n* = 3). **(B),** Quantitative PCR analysis of indicated mRNA levels in 3T3-L1-derived adipocytes transfected with siCtrl or siPTPN2 in response to TNFα (*n* = 4). **(C),** Representative Western blotting images of indicated proteins in BAT (left) and corresponding quantitative analysis (right, *n* = 5). **(D),** Immunohistochemical staining of UCP1 in BAT (*n* = 5, scale bar: 20 μm). **(E),** Western blotting analysis of indicated proteins in SAT (*n* = 5). **(F),** Immunohistochemical staining of UCP1 in SAT (*n* = 5, scale bar: 20 μm). *P<0.05 , **P<0.01 , ***P<0.001 .

As aging aggravates the loss of brown adipocytes (Rogers et al., 2012) and PTPN2 was demonstrated to regulate senescence in adipose tissue, we evaluated regulatory genes that promoted adipocyte browning. Firstly, PTPN2 was silenced in 3T3-L1-derived adipocytes and then stimulated with TNFα. The expression of specific markers including UCP1, PGC1α [proliferator-activated receptor gamma coactivator-1-alpha, which regulates mitochondrial biogenesis and fatty acid oxidation (Yan et al., 2016)], FABP4 [fatty acid-binding protein-4, which regulates adipocyte differentiation and β-oxidation of fatty acids (Walenna et al., 2018)] and INSR (insulin receptor, relating to adipocyte differentiation and function) were assessed at both of the protein (Figure 6A) and mRNA (Figure 6B) levels. The results revealed that PTPN2 knockdown inhibited the browning process of adipocytes. White adipocyte browning develops predominantly in response to cold exposure and adrenergic stress, and it has become a therapeutic target for weight gain and metabolic dysfunction (Sidossis et al., 2015; Sakers et al., 2022).

Then we assessed the influence of PTPN2 on adipose tissue browning activity *in vivo*. The protein levels of Acox1 (acyl-coenzyme A oxidase 1, which regulates β-oxidation of adipose tissue (De Carvalho et al., 2021)), INSR, PGC1α, PPARγ [peroxisome-proliferator-activated receptor-gamma, which activates the thermogenic brown fat gene program (Ohno et al., 2012)], SIRT3 [Sirtuin 3, a NAD-dependent deacetylase which regulates mitochondrial fatty-acid oxidation (Hirschey et al., 2010)], SLC27A1 [fatty acid transport protein 1, which promotes the WAT-browning process (Guo et al., 2022)] and UCP1 in adipose tissue were detected by Western blotting. Both mice and humans have thermogenic BAT. A major murine BAT depot is located in the interscapular region and is also found in cervical, axillary, perivascular, and perirenal regions. Human infants also possess BAT depot in the interscapular region, however, it later regresses and is absent in adults. Adult humans possess variable BAT and beige adipose tissue in the paravertebral junctions, cervical/axillary regions and in perirenal/adrenal locations (Sakers et al., 2022).

For BAT, our results showed that overexpressed PTPN2 promoted the protein levels of these browning-related regulators (Figure 6C). Moreover, the protein level of the best characterized thermogenic effector, UCP1, was re-confirmed by immunohistochemical staining, which indicated that overexpressed PTPN2 in T2DM + PTPN2 group facilitated the protein level of UCP1 compared with the T2DM group (Figure 6D). The same analysis was performed in SAT and the protein level of Acox1, INSR, PGC1α, PPARγ, SIRT3, SLC27A1 and UCP1 was upregulated by PTPN2 overexpression (Figures 6E, F), illustrating the role of PTPN2 in promoting the WAT-browning process.

### PTPN2 overexpression ameliorates adipose tissue functional remodeling in T2DM mice

**FIGURE 7:**
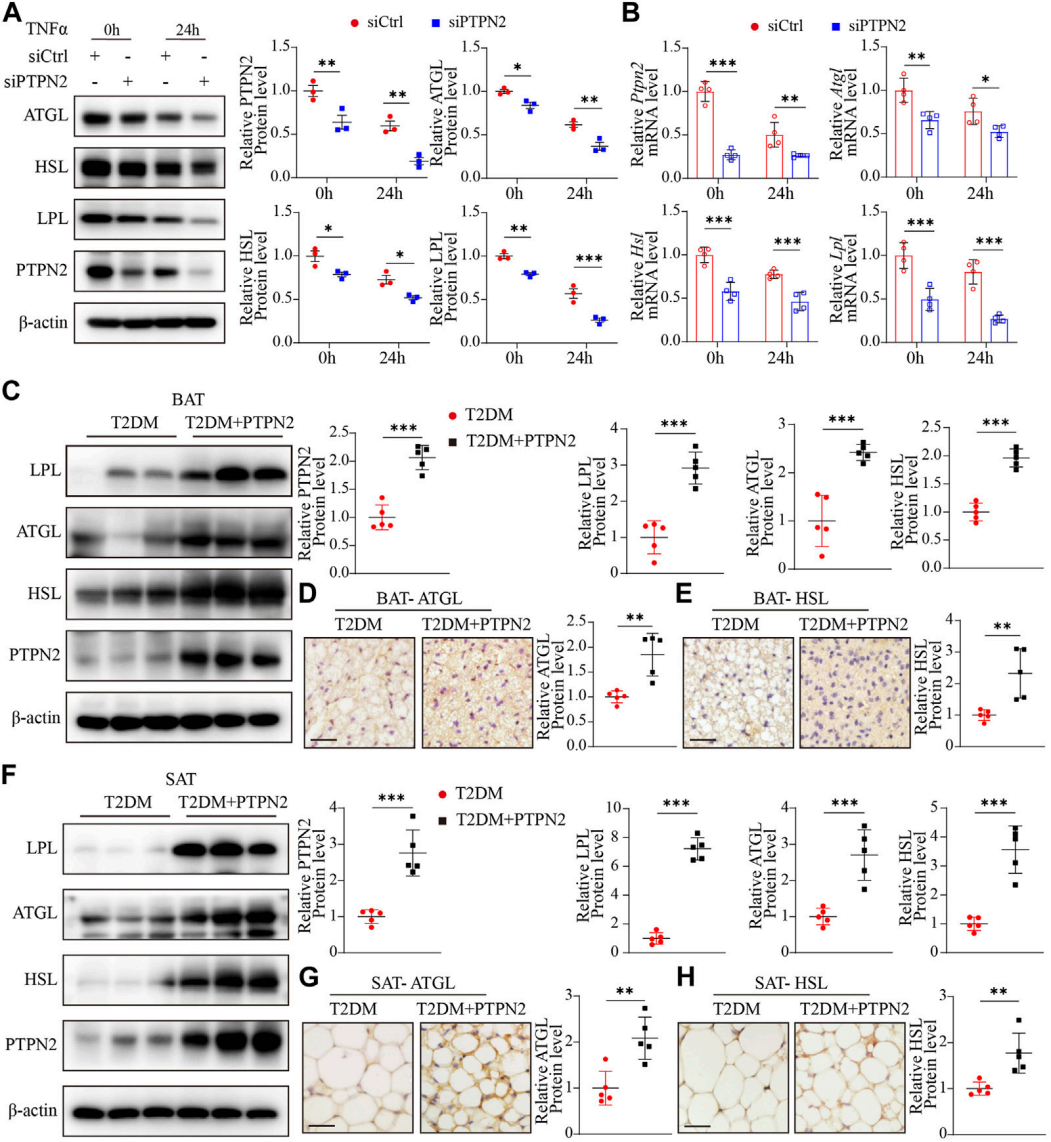
PTPN2 overexpression ameliorates adipose tissue functional remodeling in T2DM mice. **(A),** Western blotting analysis of indicated proteins in 3T3-L1-derived adipocytes transfected with siCtrl or siPTPN2 in response to TNFα (*n* = 3). **(B),** Quantitative PCR analysis of indicated mRNA levels in 3T3-L1-derived adipocytes transfected with siCtrl or siPTPN2 with TNFα stimulation (*n* = 4). **(C),** Representative Western blotting images of indicated proteins in BAT (left) and corresponding quantitative analysis (right, *n* = 5). **(D, E),** Immunohistochemical staining of ATGL **(D)** and HSL **(E)** in BAT, respectively (*n* = 5, scale bar: 20 μm). **(F),** Western blotting analysis of indicated proteins in SAT (*n* = 5). **(G, H),** Immunohistochemical staining of ATGL **(G)** and HSL **(H)** in SAT (*n* = 5, scale bar: 20 μm). *P<0.05 , **P<0.01 , ***P<0.001 .

Adipose tissue remodeling is a complex process and is regulated by various metabolic challenges. Adipocytes have the ability to act rapidly in response to excess caloric intake and hypertrophy and hyperplasia are the main mechanisms of adipocyte plasticity (Zhu et al., 2022). Adipose tissue remodeling can be pathologically accelerated in the obese state, featuring reduced angiogenic remodeling, a heightened state of immune cell infiltration and subsequent proinflammatory responses (Sun et al., 2011). Disturbance of lipid metabolism contributes to the progression of IR in T2DM, so we investigated the role of PTPN2 in controlling lipid metabolism. Experiments *in vitro* were performed using 3T3-L1-derived adipocytes transfected with PTPN2-siRNA, and lipid metabolic gene expression was analyzed at the protein and mRNA levels. The results of Western blotting demonstrated that the levels of ATGL (adipose triglyceride lipase, which hydrolyzes intracellular triglyceride), HSL (hormone-sensitive lipase, which is involved in lipid catabolism (Jocken et al., 2016)) and LPL (lipoprotein lipase, which catalyzes the hydrolysis of triglyceride-rich lipoproteins (Jocken et al., 2016)) was reduced in PTPN2-silent 3T3-L1-derived adipocytes compared with the control group (Figures 7A, B).

Then we assessed the effect of PTPN2 on lipid homeostasis in adipose tissue *in vivo*. Compared with the T2DM group, the protein levels of LPL, ATGL and HSL was significantly higher in BAT in T2DM + PTPN2 group (Figures 7C–E). Consistently, these regulators hydrolyzing triglycerides and promoting thermogenesis were upregulated with PTPN2 overexpression in SAT (Figures 7F–H), confirming the function of PTPN2 on preventing lipid retention in adipocytes.

## Discussion

As a crucial metabolic organ for energy homeostasis, adipose tissue is critical in regulating IR through its lipid storage capacity, thermogenic role and endocrine functions. Energy imbalance due to excess lipid retention results in adipocyte hypertrophy (an increase in adipocyte size) and WAT remodeling, especially for SAT (Bays, 2014). As a key thermogenic protein in BAT, UCP1 plays an essential role in energy expenditure, which can be regulated by many environmental factors like diet (Okla et al., 2017). Our study observed that in a model of T2DM, adipocyte hypertrophy with more lipids was significant in SAT and BAT, which was reversed by PTPN2 overexpression. Besides, the protein levels of UCP1 and other indicators promoting energy consumption was also upregulated by PTPN2. Therefore, IR could be improved by PTPN2 via shrinking white adipocyte size, reducing lipid retention and enhancing energy expenditure. Free fatty acids, which were derived from hydrolysis of triglycerides, act as fuel substrate for thermogenesis (Schreiber et al., 2017). Furthermore, we observed that proteins facilitating hydrolysis of triglycerides including ATGL and HSL were upregulated by PTPN2 overexpression, which confirmed the role of PTPN2 in promoting lipid decomposition and energy expenditure. Although PTPN2 has been previously reported to be involved in adipose tissue browning indirectly, our study elucidated its role in regulating the browning process of adipocytes directly for the first time.

Senescent cells have been found in adipose tissue of a T2DM state and aging has been reported to promote IR (Lee et al., 2022). Adipose tissue browning is inhibited during aging (Berry et al., 2017), with a functional decline of mitochondria and UCP1 activation. Meanwhile, the potential of progenitor cells for beige adipogenesis declines due to senescence, therefore, the reduction of UCP1 protein level in WAT and impaired lipid mobilization were induced in T2DM (Koenen et al., 2021). Our study suggested that PTPN2 overexpression lowered senescence-related factors like P16 and P53 in adipose tissue under a T2DM state. As PTPN2 expression is downregulated in T2DM (Zheng et al., 2018), we proposed that PTPN2 at the crossroad of adipose tissue browning, lipid disturbance and IR might be involved in senescence of adipocytes in T2DM. Adipocytes are the primary cell type in adipose tissue exhibiting the role of energy reservoir and endocrine function (Lee et al., 2022). However, adipocyte senescence still needs to be further studied. Our study *in vitro* emphasized that senescence in adipocytes might impede the browning process of adipocytes and we first demonstrated that PTPN2 improved adipocyte senescence from the state of preadipocytes, thus promoting the browning process.

The MAPK/NF-κB pathway mediates signaling to various biological processes including cell senescence. Berry et al. (Berry et al., 2017) showed that phosphorylation of p38/MAPK contributed to p16 expression while dephosphorylation improved senescence of adipocytes. The activation of TAK1 is involved in the activation of downstream kinases including MAPK/NF-κB identified in cellular senescence (Rajagopalan et al., 2014). Our study is the first to investigate that PTPN2 could interact with TAK1 directly and catalyze dephosphorylation of TAK1 in adipocytes, resulting in dephosphorylation of the downstream proteins (Erk, P38, JNK, IκB, and P65) subsequently. Thus, TAK1 exhibited a role by which PTPN2 ameliorated cellular senescence and facilitated the browning process of adipocytes.

In summary, our study revealed that PTPN2 gene overexpression promoted adipose tissue browning by attenuating senescence, thus improving glucose tolerance and IR in T2DM mice. Mechanistically, we are the first to demonstrate that PTPN2 targeted TAK1 directly for dephosphorylation to inhibit the downstream MAPK/NF-κB pathway and ameliorated cellular senescence in adipocytes consequently. Therefore, PTPN2 in adipocytes may be a potential therapeutic target for T2DM.

### Limitation

The recombinant pAdxsi adenovirus system we used in this study is not specifically expressed on adipose tissue so the non-specific expression in other tissues may influence the regulation of glucose and lipid metabolism. Further studies need to be conducted in the future to determine the function of PTPN2 in adipocytes *in vivo* using gene conditional knockout mice or recombinant pAdxsi adenovirus specifically expressed.

## Data Availability

The original contributions presented in the study are included in the article/Supplementary Material, further inquiries can be directed to the corresponding authors.
