# Supplementary figures and images for: PTPN2 targets TAK1 for dephosphorylation to improve cellular senescence and promote adipose tissue browning in T2DM
Source: Front Pharmacol. 2023 May 12;14:1124633. doi: 10.3389/fphar.2023.1124633 (PMC10213551; doi:10.3389/fphar.2023.1124633)

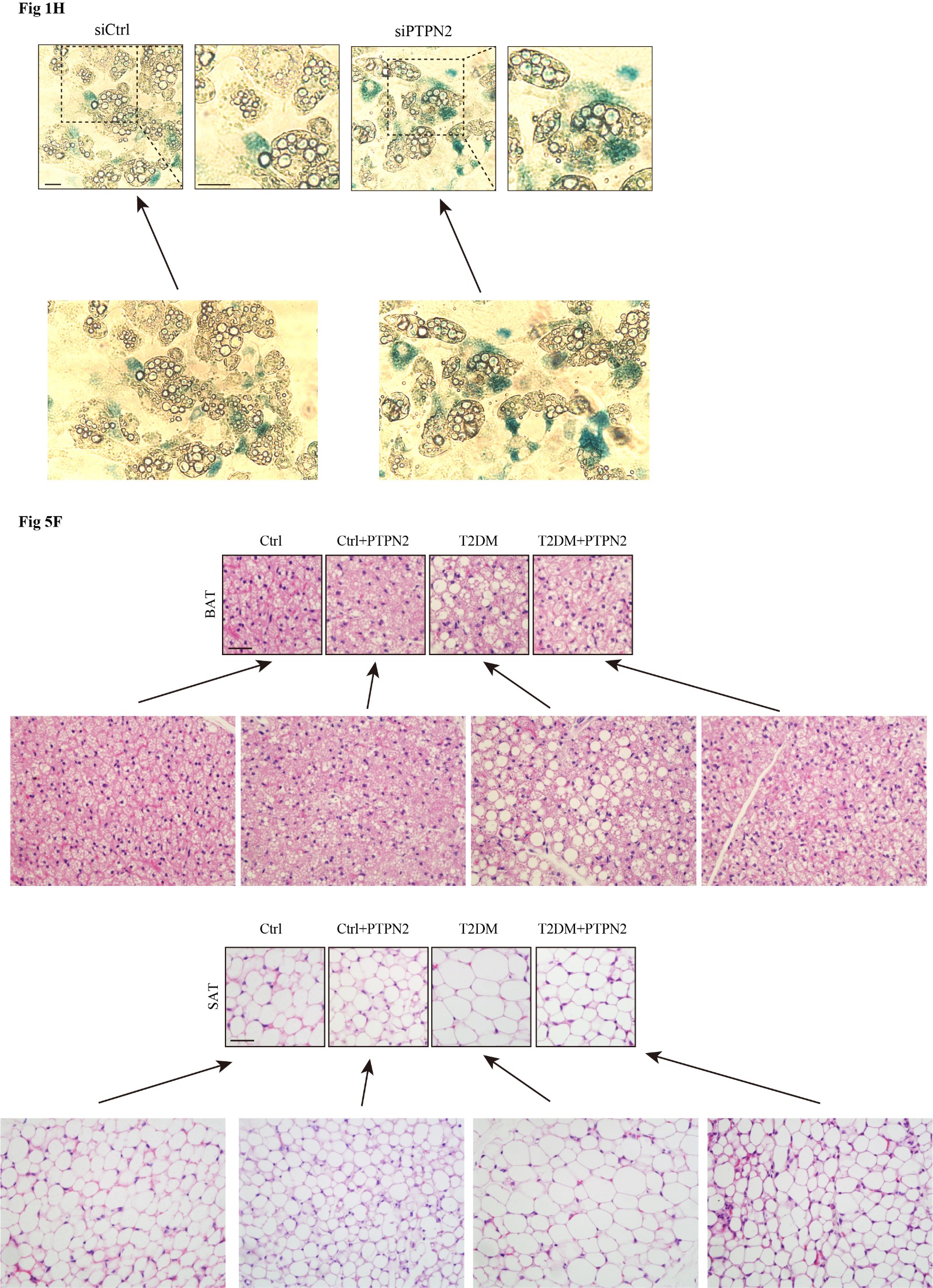


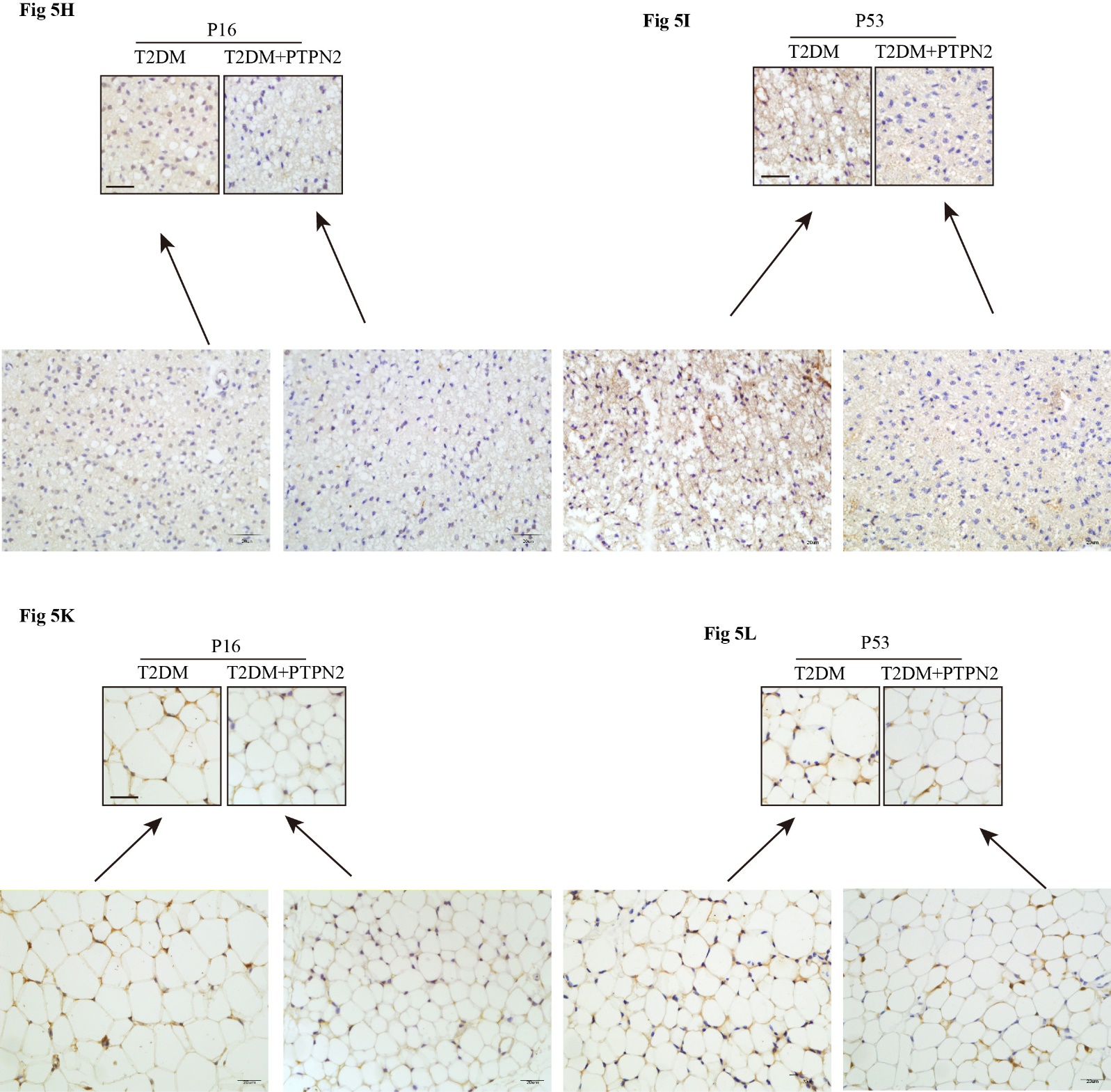


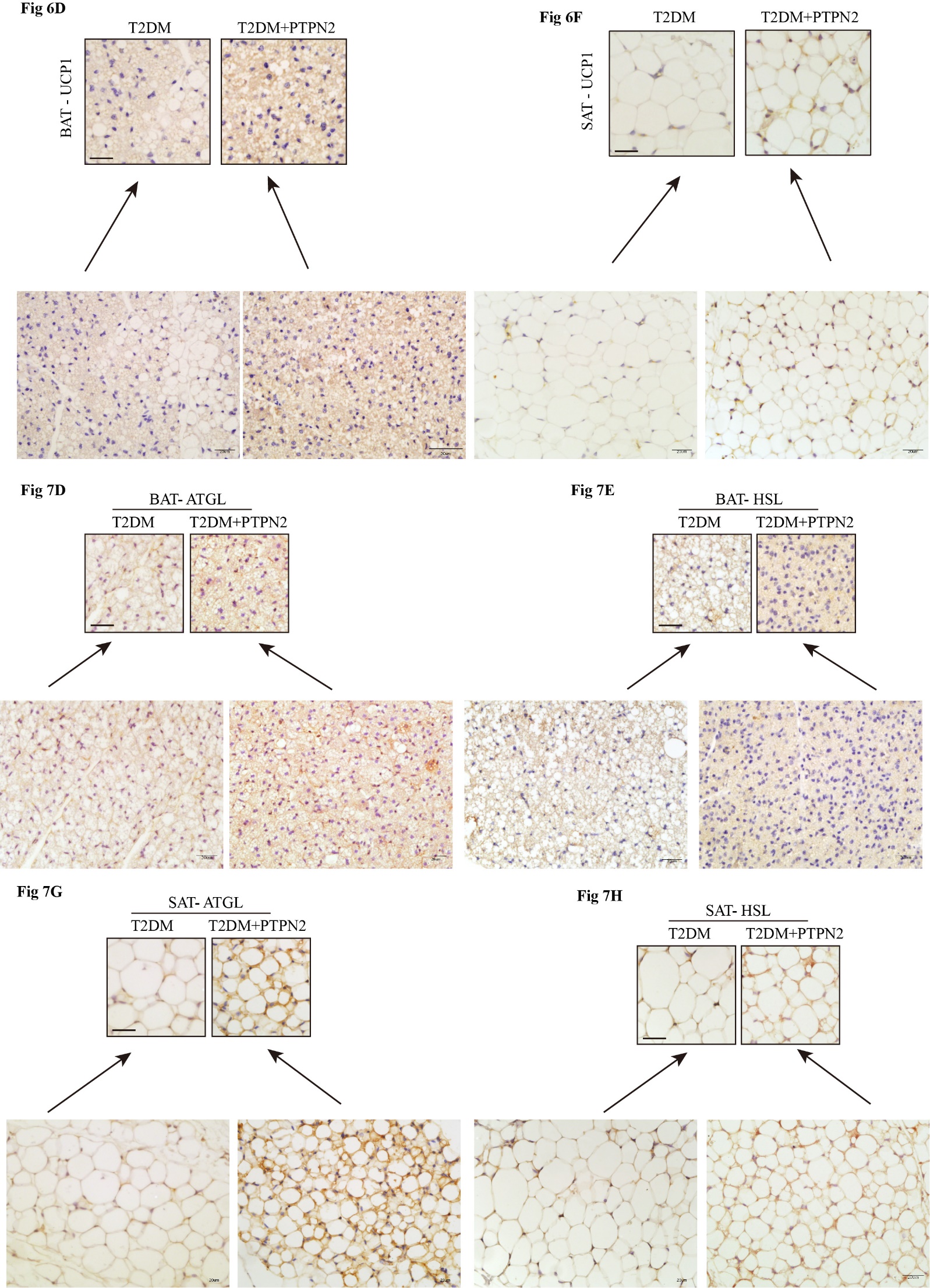


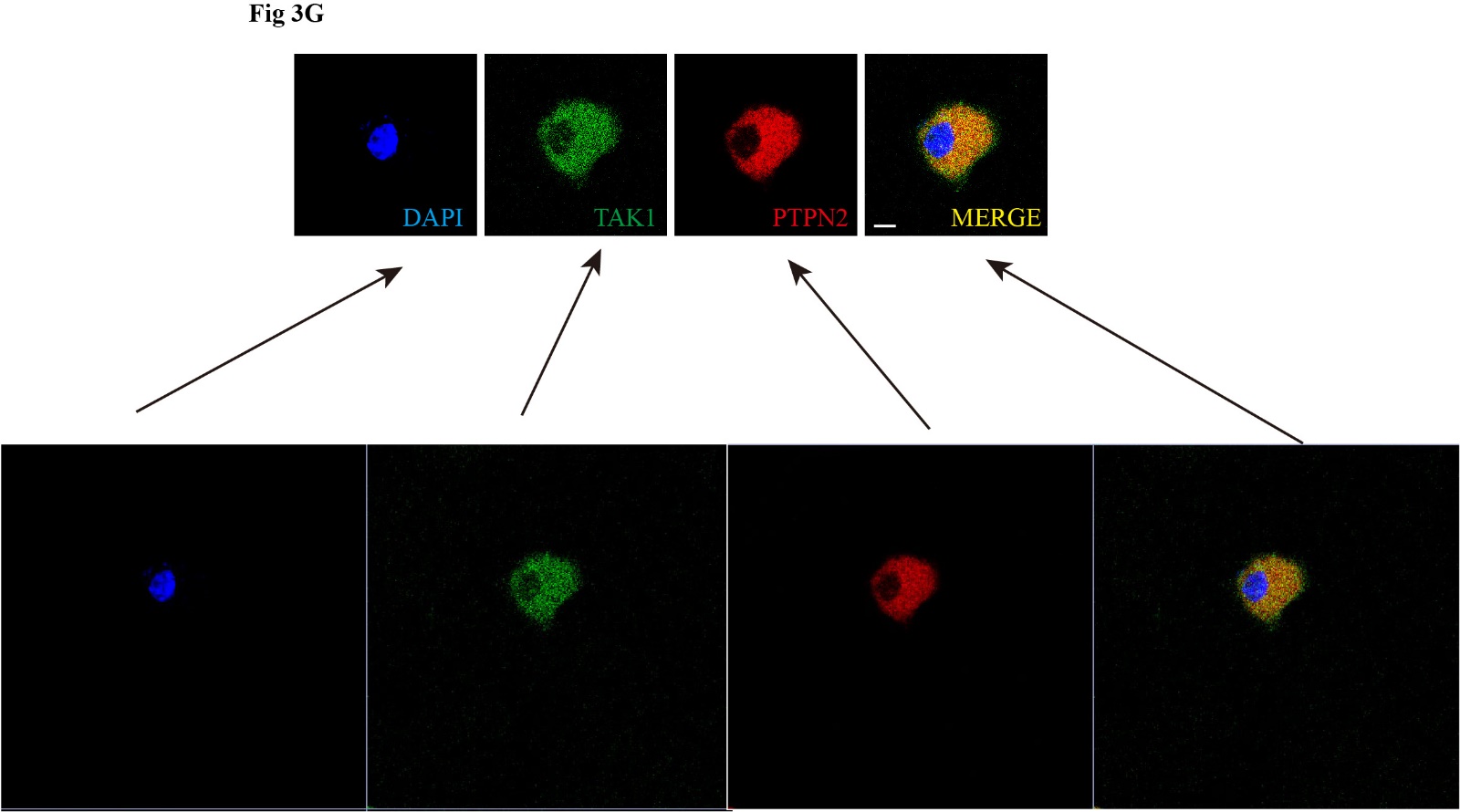

Supplement: Supplementary file 2 [file Table3.DOCX]

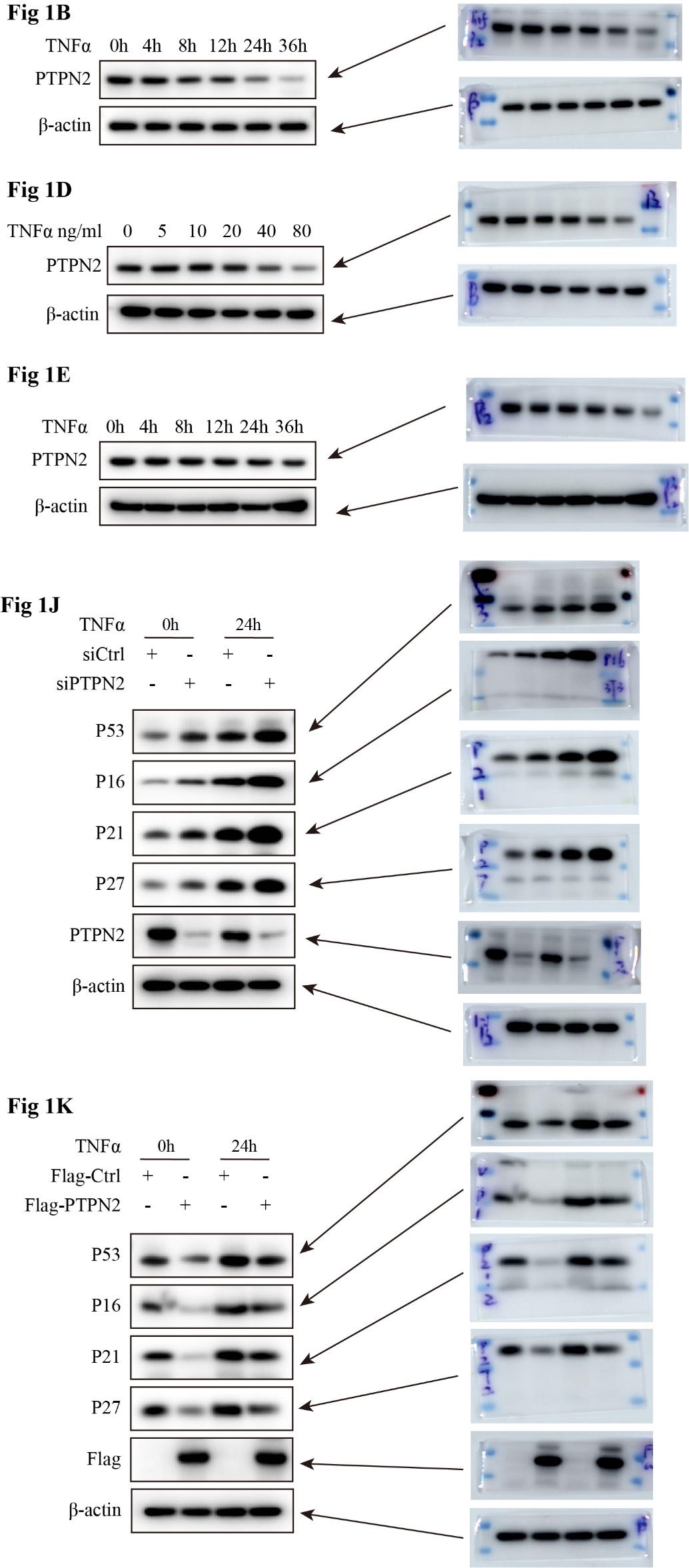

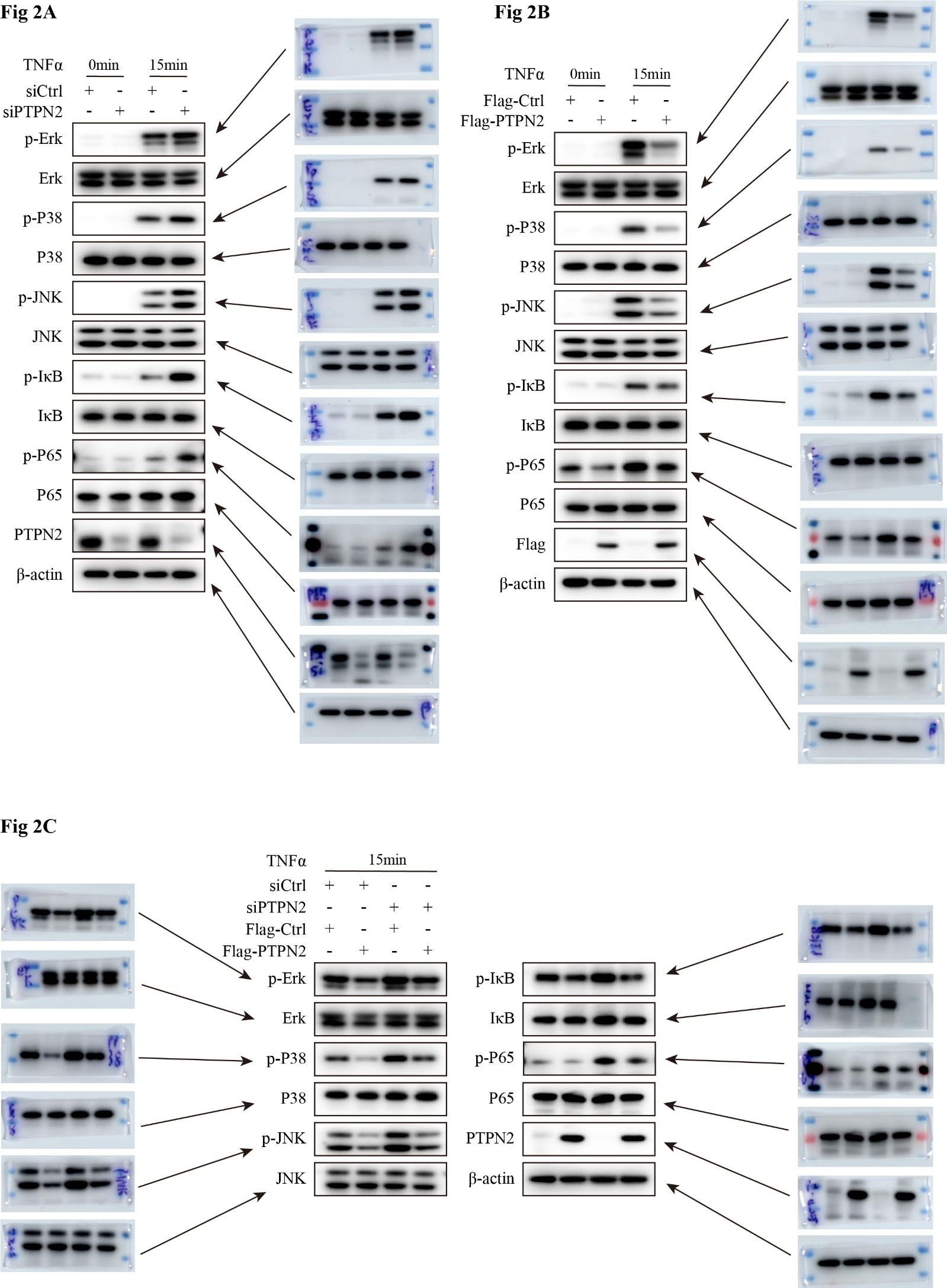


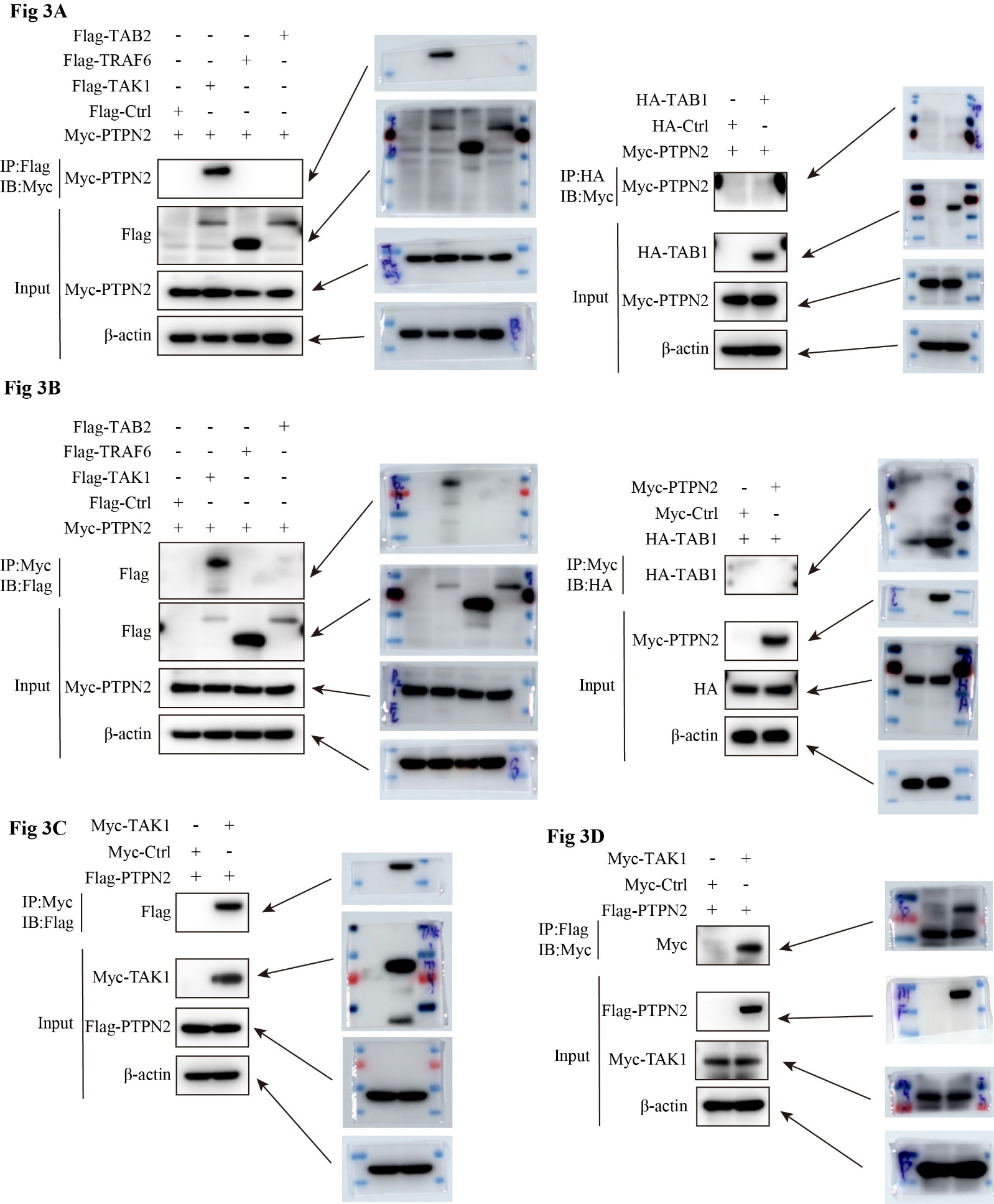


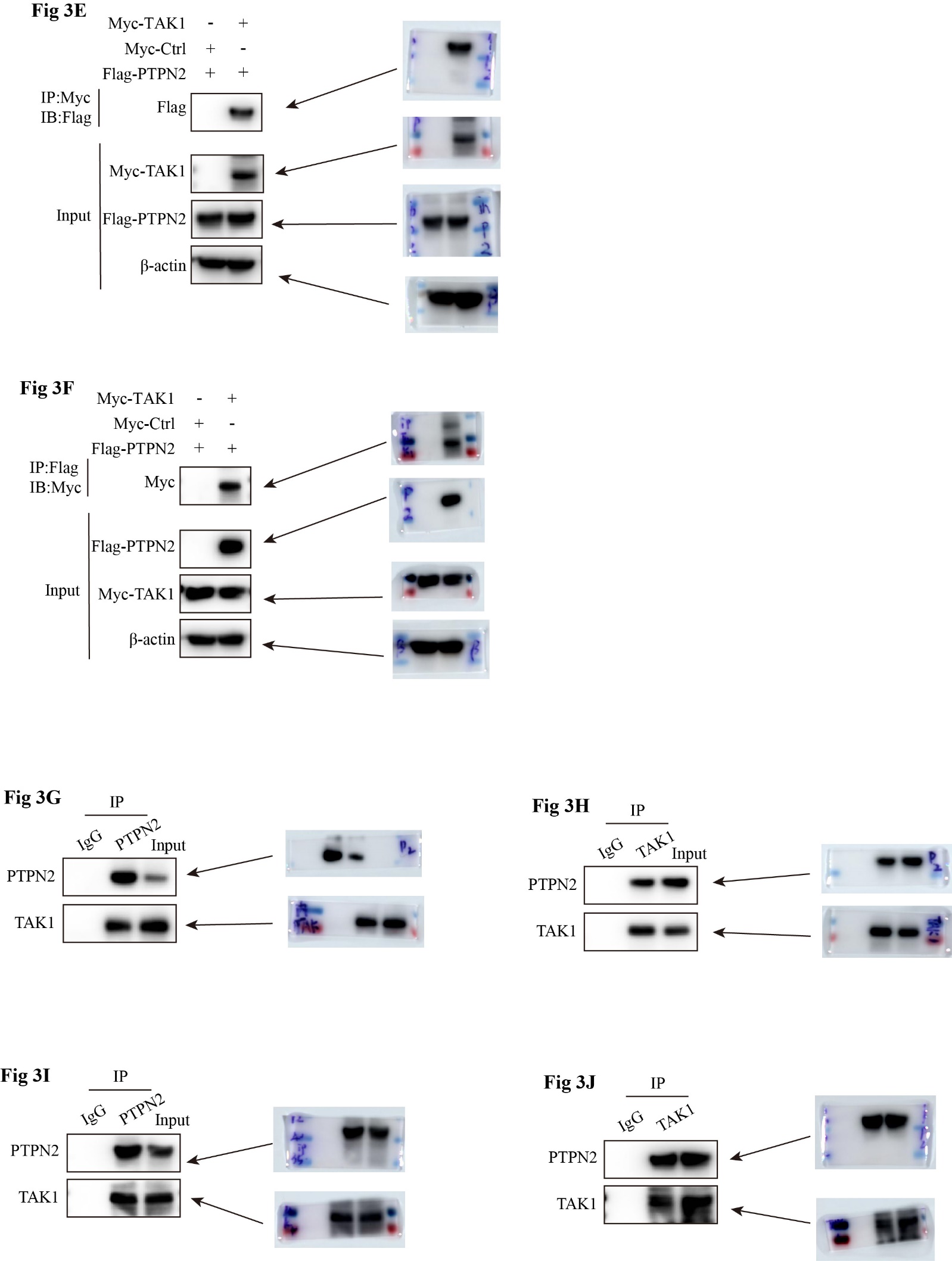


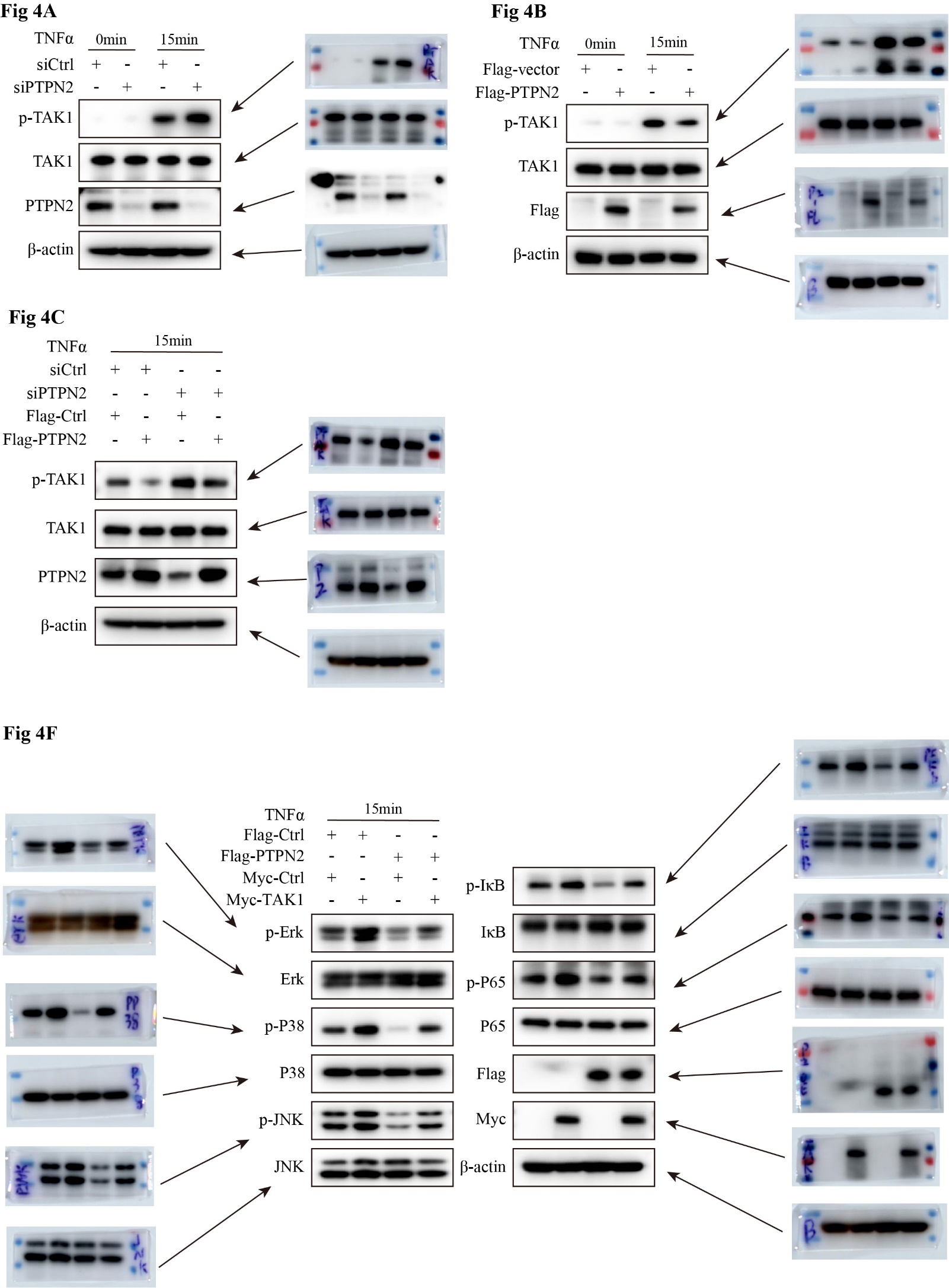

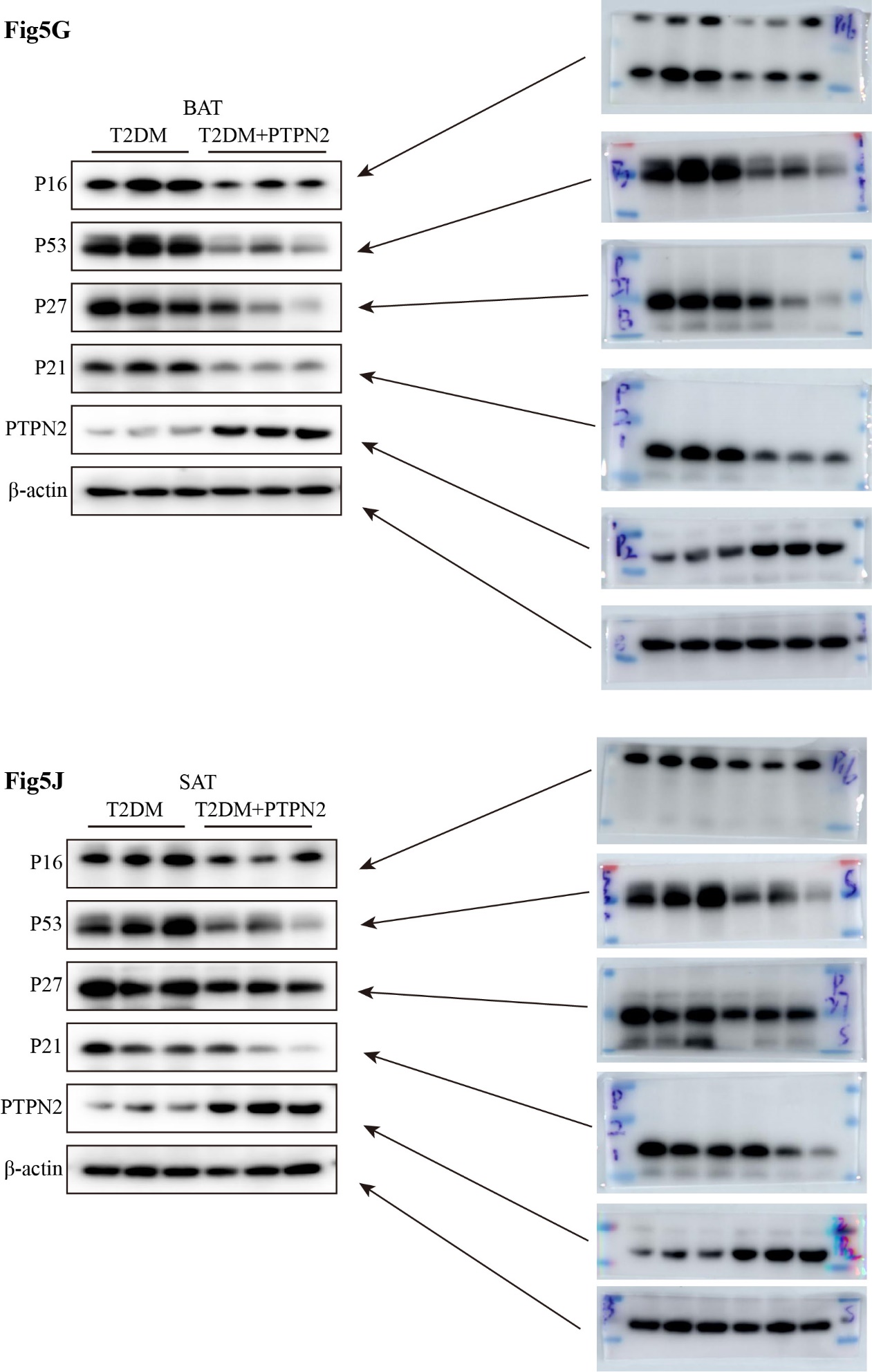


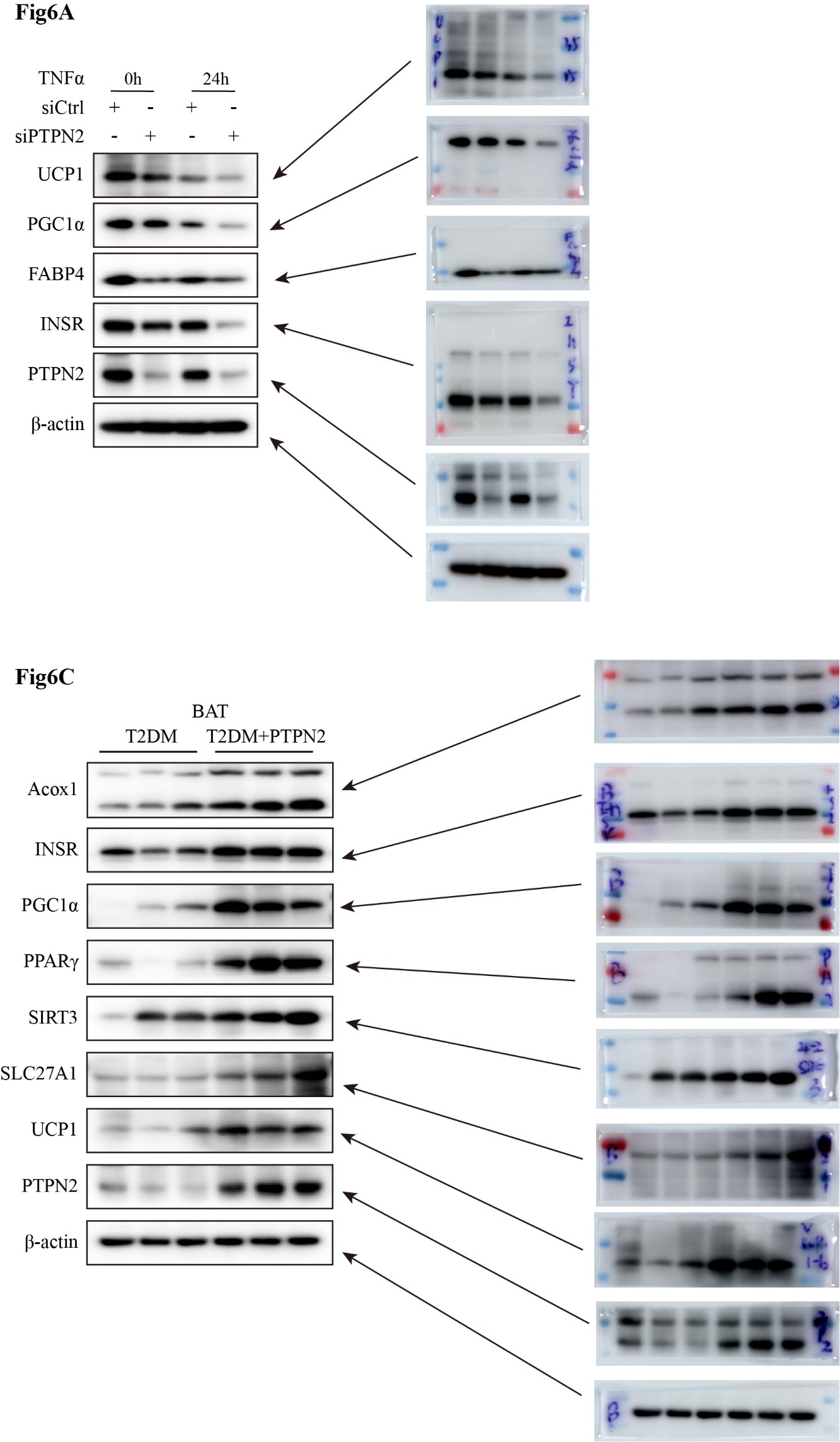


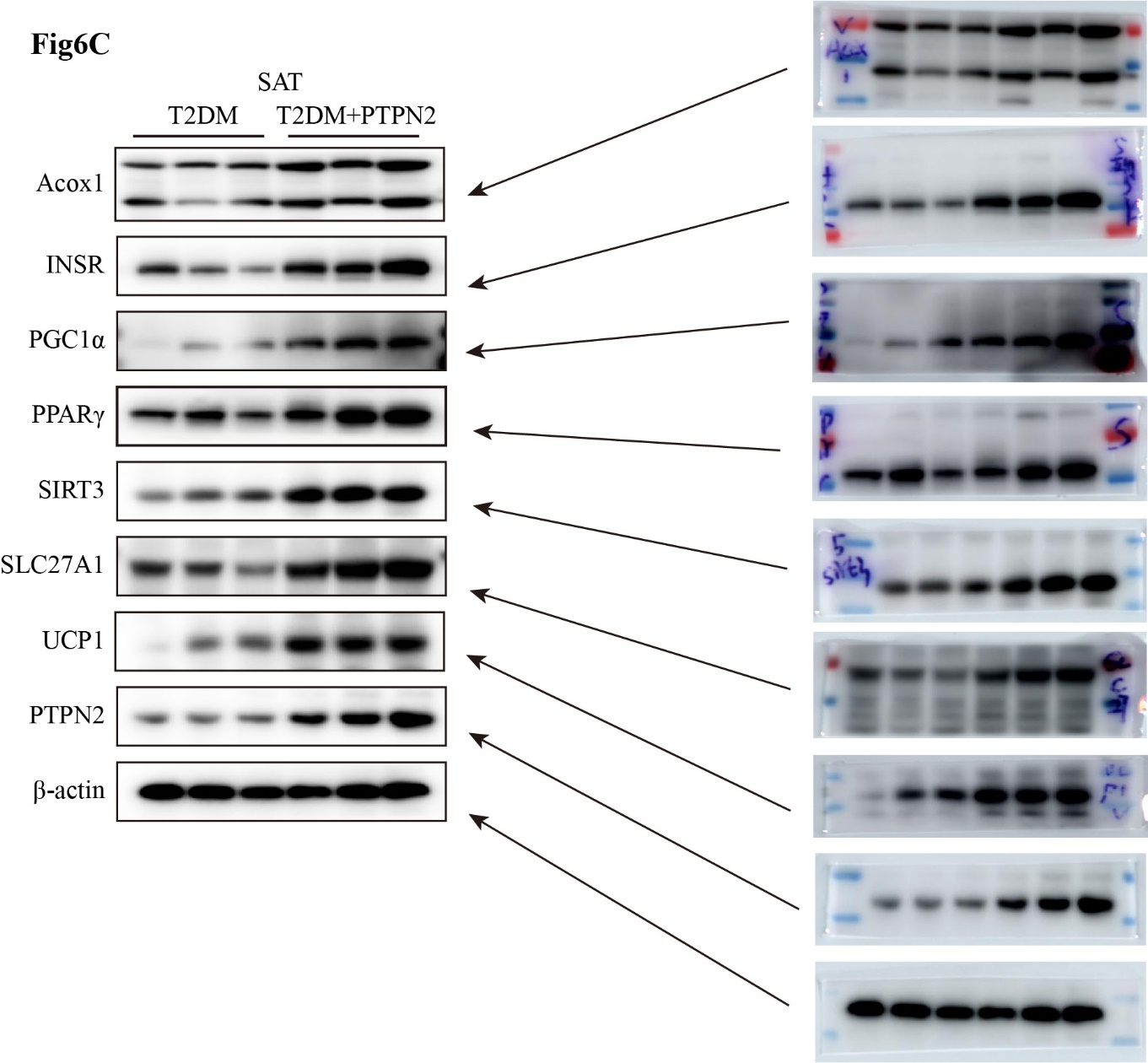


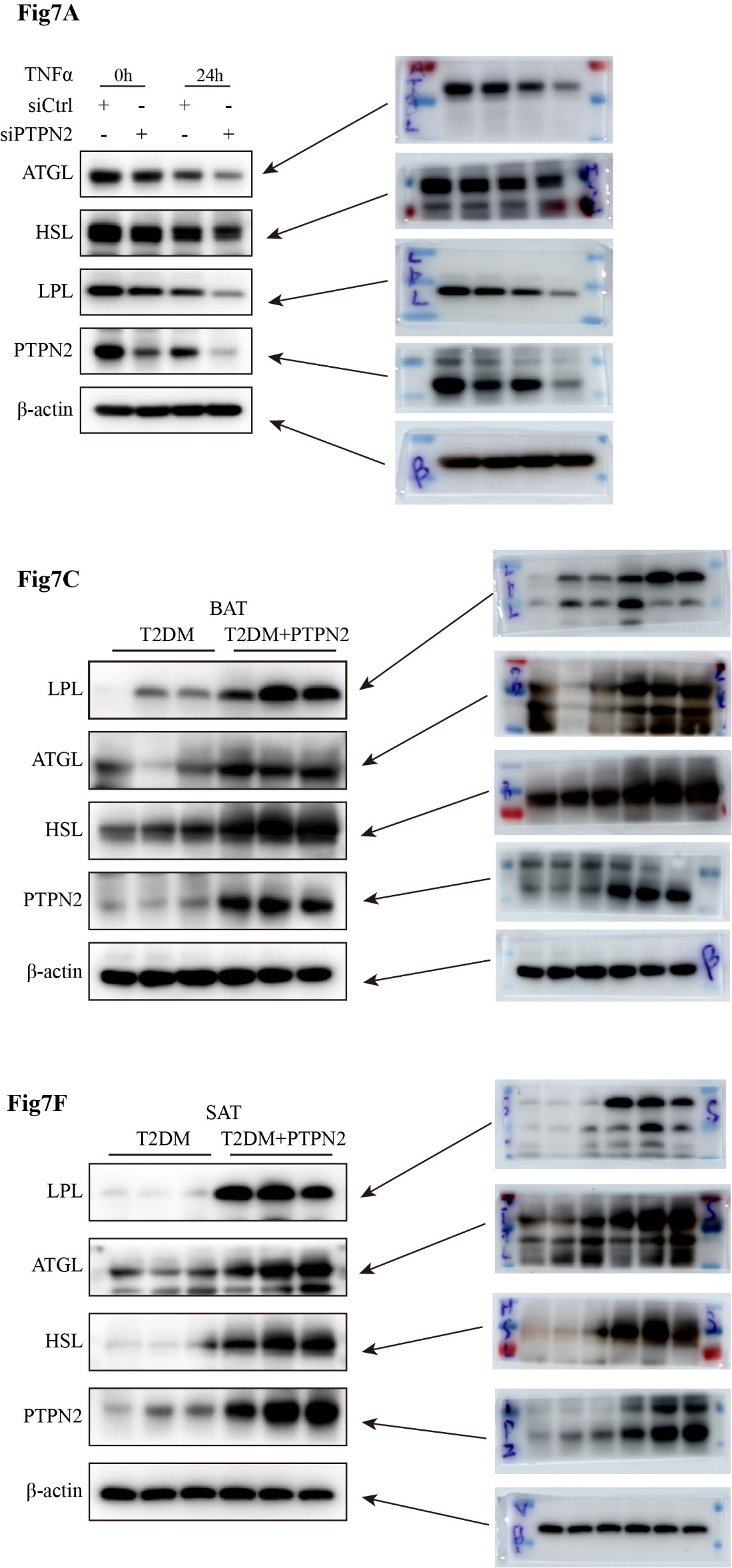

Supplement: Supplementary file 4 [file Table4.DOCX]
